# Supplementary material for: Human papillomavirus seroprevalence in pregnant women following gender-neutral and girls-only vaccination programs in Finland: A cross-sectional cohort analysis following a cluster randomized trial
Source: PLoS Med. 2021 Jun 7;18(6):e1003588. doi: 10.1371/journal.pmed.1003588 (PMC8216524; doi:10.1371/journal.pmed.1003588)
Supplement: S1 Fig — (a) Among females; (b) among males. (DOCX) [file pmed.1003588.s002.docx]

**
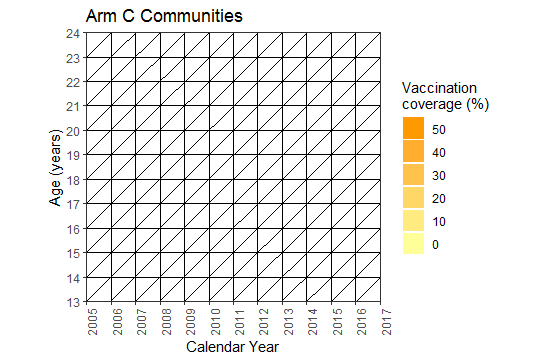

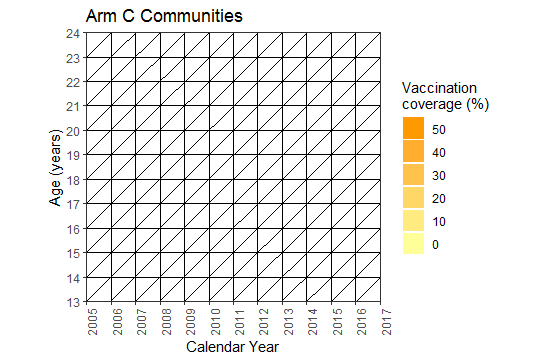

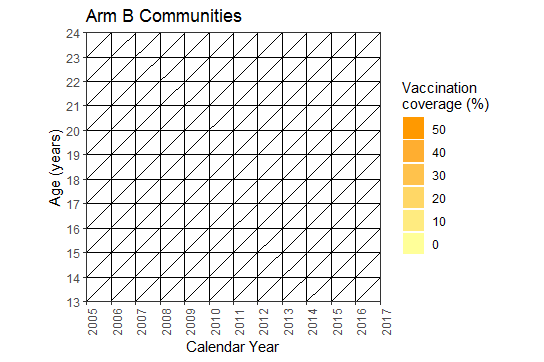

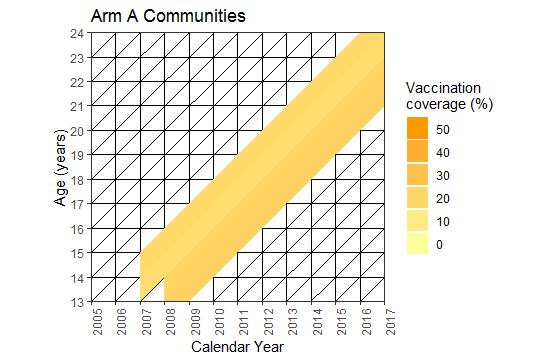

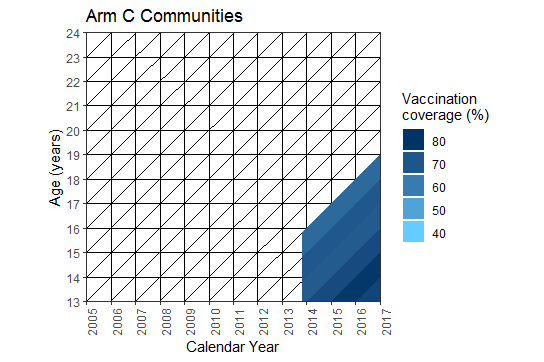

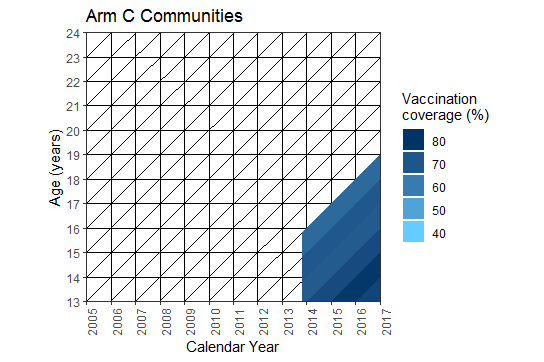

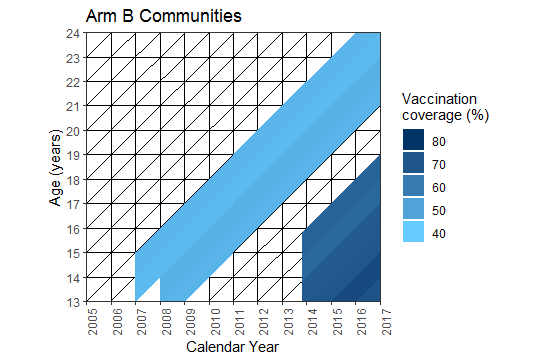

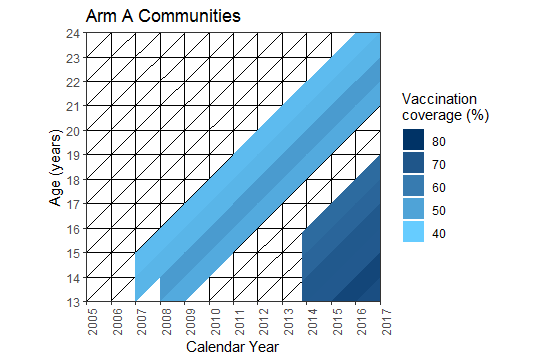
S1 Fig:** Lexis diagrams depicting the vaccinated cohorts and vaccination coverage among the eligible birth cohorts of the study population, by Arm, and gender ([a] among females and [b] among males).

**b)**

**a)**
