# Supplementary material for: Human papillomavirus seroprevalence in pregnant women following gender-neutral and girls-only vaccination programs in Finland: A cross-sectional cohort analysis following a cluster randomized trial
Source: PLoS Med. 2021 Jun 7;18(6):e1003588. doi: 10.1371/journal.pmed.1003588 (PMC8216524; doi:10.1371/journal.pmed.1003588)
Supplement: S2 Fig — (DOCX) [file pmed.1003588.s003.docx]

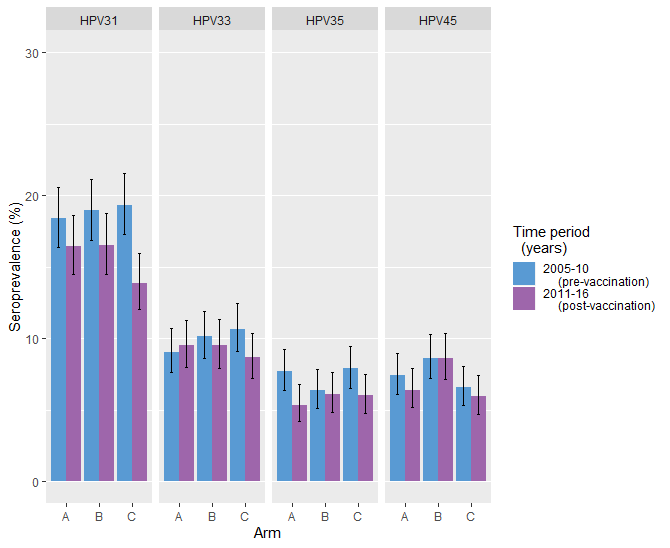
**S2 Fig:** Type-specific human papillomavirus (HPV) seroprevalence (%) among unvaccinated females under the age of 23 years-old by vaccination strategy: gender-neutral (Arm A), girls-only (Arm B) and control (Arm C), and time period of sample donation (pre-vaccination era, 2005-2010, and post-vaccination era, 2011-2016).
