# Supplementary material for: Human papillomavirus seroprevalence in pregnant women following gender-neutral and girls-only vaccination programs in Finland: A cross-sectional cohort analysis following a cluster randomized trial
Source: PLoS Med. 2021 Jun 7;18(6):e1003588. doi: 10.1371/journal.pmed.1003588 (PMC8216524; doi:10.1371/journal.pmed.1003588)
Supplement: S3 Fig — Arm-specific PRs comprise post-vaccination to pre-vaccination-era HPV seroprevalence ratios among pregnant unvaccinated Finnish females aged under 23 years old, and are adjusted for community-level maternal smoking. (DOCX) [file pmed.1003588.s004.docx]

***
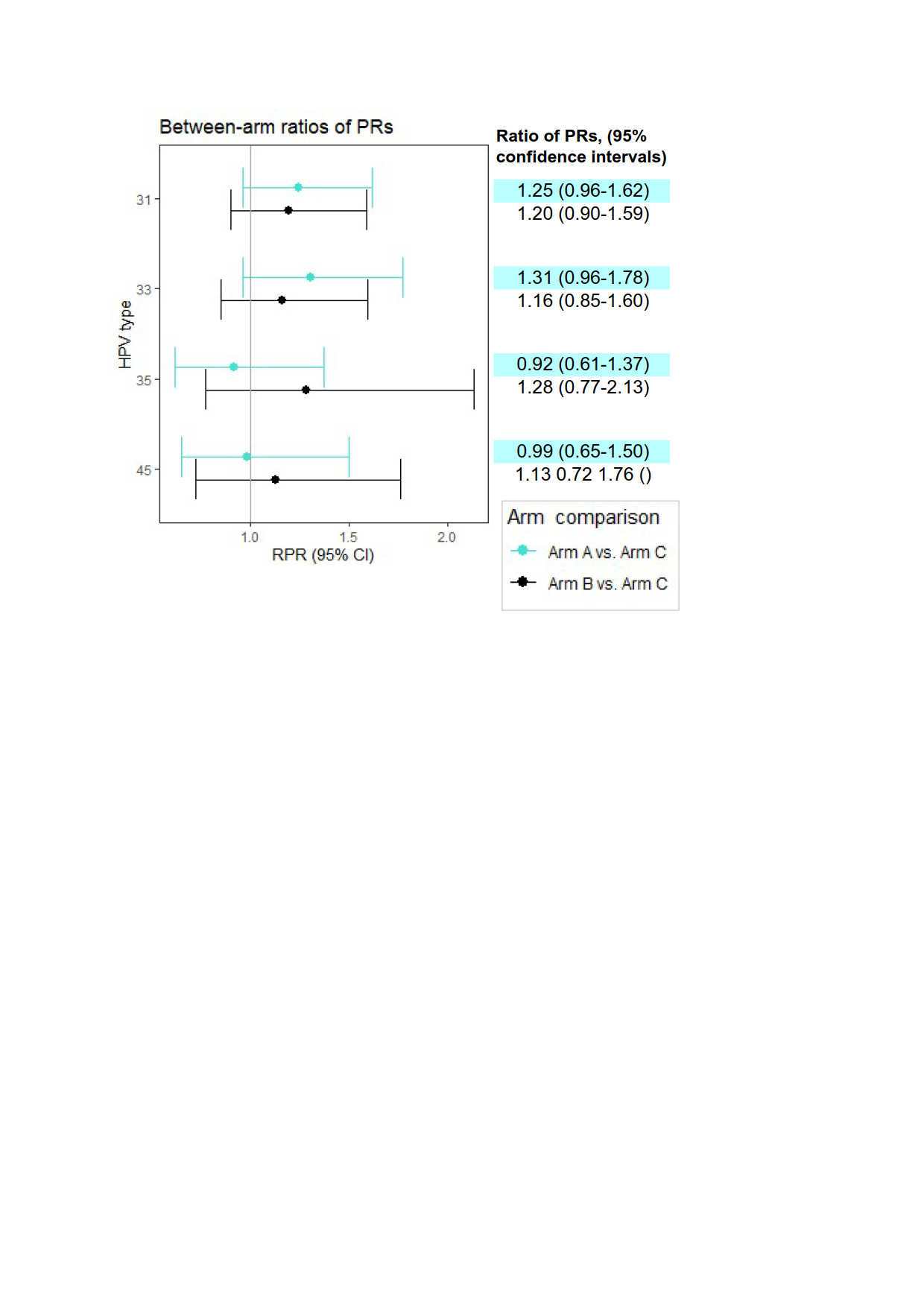
*S3 Fig:** Ratio of HPV seroprevalence ratios (RPR) comparing Arm A/B to Arm C. Arm specific PRs comprise of post-vaccination to pre-vaccination era HPV seroprevalence ratios among pregnant unvaccinated Finnish females, aged under 23 years old, and are adjusted for community-level maternal smoking.
