# Supplementary material for: Human papillomavirus seroprevalence in pregnant women following gender-neutral and girls-only vaccination programs in Finland: A cross-sectional cohort analysis following a cluster randomized trial
Source: PLoS Med. 2021 Jun 7;18(6):e1003588. doi: 10.1371/journal.pmed.1003588 (PMC8216524; doi:10.1371/journal.pmed.1003588)
Supplement: S1 Table — (DOCX) [file pmed.1003588.s005.docx]

**Table S1:** Sensitivity and specificity parameters of pseudovirion-based serology in measuring cumulative HPV exposure used in the probabilistic bias analysis.
