# Supplementary material for: Human papillomavirus seroprevalence in pregnant women following gender-neutral and girls-only vaccination programs in Finland: A cross-sectional cohort analysis following a cluster randomized trial
Source: PLoS Med. 2021 Jun 7;18(6):e1003588. doi: 10.1371/journal.pmed.1003588 (PMC8216524; doi:10.1371/journal.pmed.1003588)
Supplement: S2 Table — HSV-2, herpes simplex virus type 2. (DOCX) [file pmed.1003588.s006.docx]

**Table S2:** Intracluster correlation coefficient (ICC) of any human papillomavirus (HPV) type seropositivity among pregnant females donating sera during the pre-vaccination era from 2005-2010. HSV-2= herpes simplex virus type 2.
