# Supplementary material for: Human papillomavirus seroprevalence in pregnant women following gender-neutral and girls-only vaccination programs in Finland: A cross-sectional cohort analysis following a cluster randomized trial
Source: PLoS Med. 2021 Jun 7;18(6):e1003588. doi: 10.1371/journal.pmed.1003588 (PMC8216524; doi:10.1371/journal.pmed.1003588)
Supplement: S4 Table — (DOCX) [file pmed.1003588.s008.docx]

**Table S4:** Absolute HPV type specific seroprevalence among unvaccinated pregnant Finnish stratified by a) trial Arm and vaccination era (2005-10 is defined as the ‘pre-vaccination era’ and 2011-16 as the ‘post-vaccination era’), and b) additionally by HSV-2 seropositivity.
