# Supplementary material for: Human papillomavirus seroprevalence in pregnant women following gender-neutral and girls-only vaccination programs in Finland: A cross-sectional cohort analysis following a cluster randomized trial
Source: PLoS Med. 2021 Jun 7;18(6):e1003588. doi: 10.1371/journal.pmed.1003588 (PMC8216524; doi:10.1371/journal.pmed.1003588)
Supplement: S5 Table — Comparisons are between 2 time periods of sample donation (2011–2016, post-vaccination era, versus 2005–2010, pre-vaccination era), stratified by intervention Arm A (gender-neutral HPV vaccination), Arm B (girls-only HPV vaccination), and Arm C (control vaccination). (DOCX) [file pmed.1003588.s009.docx]

**Table S5:** Unadjusted HPV type specific seroprevalence ratio (PR) among unvaccinated Finnish females comparing the post-vaccination era to the pre-vaccination era. Comparisons are between two time periods of sample donation (2011-2016, post-vaccination era vs. 2005-2010, pre-vaccination era), stratified by intervention Arm A (gender-neutral HPV vaccination), Arm B (girls only HPV vaccination) and Arm C (control vaccination).
