# Supplementary material for: Human papillomavirus seroprevalence in pregnant women following gender-neutral and girls-only vaccination programs in Finland: A cross-sectional cohort analysis following a cluster randomized trial
Source: PLoS Med. 2021 Jun 7;18(6):e1003588. doi: 10.1371/journal.pmed.1003588 (PMC8216524; doi:10.1371/journal.pmed.1003588)
Supplement: S6 Table — All estimates are adjusted for smoking. na, not available. (DOCX) [file pmed.1003588.s010.docx]

**Table S6:** Adjusted seroprevalence ratio (PR) of HPV seropositivity by HPV type among pregnant, unvaccinated Finnish females under the age of 23 years old by study Arm (gender neutral vaccination Arm A, females only vaccination Arm B or control Arm C), comparing time period of sample donation (post-vaccination era, 2011-2016, compared to the 2005-2010, pre-vaccination era), and stratified by herpes simplex virus type-2 serostatus. All estimates are adjusted for smoking. *na, not available.
