# Supplementary material for: Human papillomavirus seroprevalence in pregnant women following gender-neutral and girls-only vaccination programs in Finland: A cross-sectional cohort analysis following a cluster randomized trial
Source: PLoS Med. 2021 Jun 7;18(6):e1003588. doi: 10.1371/journal.pmed.1003588 (PMC8216524; doi:10.1371/journal.pmed.1003588)
Supplement: S1 Text — (DOCX) [file pmed.1003588.s011.docx]

**Supplementary Methods**

*Laboratory Analysis- Extension of the assay panel*

We extended the assay panel with HPV51, 66 and HSV-2. Similarly to our previously validated HPV antigens, we produced the HPV51 and HPV66 pseudoviruses by transfecting the 293TT cell line with plasmids encoding the L1 and L2 HPV structural genes ([www.addgene.org](http://www.addgene.org), AddgenepVITRO plasmid constructs, HPV51 Cat#52583 and HPV66 Cat#52602) of a corresponding HPV type.^1^.HSV glycoprotein G (gG2) antigen and HSV-2 quality control sera were obtained from the commercially available sources (The Native Antigen Company, product code “HSV-gG2-100” and National Institute for Biological Standards and Control, QCRHSV2QC1-Anti-HSV-2 Quality Control Reagent Sample 1, code: 13/B642). HSV gG2 antigens were bound to the heparin-coated beads following the same protocol as was used for all of the HPV antigens.

We validated HSV-2 with serum samples from women previously tested for HSV-2 with a HSV-2 glycoproteingG-2–based ELISA (Biokit) (N=52).^2^ The newly introduced HSV-2 antigen was shown to be both sensitive and specific (sensitivity: HSV-2=84.6%; specificity: HSV-2=88.5%). At the beginning and at the end of the study, the extended HPV+HSV-2 panel passed the routine quality control with the positive sera panel consisting of International and in-house monospecific and multipositive standards. These standard sera are obtained either from the non-vaccinated subjects having types-specific HPV infection-induced antibodies or from the individuals who were vaccinated against HPV.

Seropositivity cut-off levels were established with a negative control panel of serum samples from 191 children ≤12 years old (average age, 4.7 years). For each antigen, the cut-offs were assigned as described in the World Health Organization HPV Laboratory Manual (i.e., by calculating the average of median fluorescence intensities [MFIs] of a negative control serum panel +3 SDs).^3^

*Statistical Analyses*

All statistical analyses were conducted using R statistical software package version 3.6.0. with the geepack package (version 1.2-1), grid package (version 3.4.3), gridExtra package (version 2.3), the DescTools package (version 0.99.28), the ggplot2 package (version 3.2.0), the ICCbin package (version 1.1.1), and the episensr package (version 0.9.4) (The R Foundation; https://www.r-project.org/).

*References*

1. Faust H, Jelen MM, Poljak M, Klavs I, Učakar V, Dillner J. Serum antibodies to human papillomavirus (HPV) pseudovirions correlate with natural infection for 13 genital HPV types. J ClinVirol 2013; 56:336-41.

2. Arnheim-Dahlstrom L, Andersson K, Luostarinen T, et al. Prospective seroepidemiological study of human papillomavirus and other risk factors in cervical cancer. Cancer Epidemiol Biomarkers Prev 2011; 20:2541-50.

3. Eklund C, Unger ER, Nardelli-Haefliger D, Zhou T, Dillner J. International collaborative proficiency study of Human Papillomavirus type 16 serology. Vaccine 2012; 30:294-9.
