# Supplementary material for: Human papillomavirus seroprevalence in pregnant women following gender-neutral and girls-only vaccination programs in Finland: A cross-sectional cohort analysis following a cluster randomized trial
Source: PLoS Med. 2021 Jun 7;18(6):e1003588. doi: 10.1371/journal.pmed.1003588 (PMC8216524; doi:10.1371/journal.pmed.1003588)

Human papillomavirus type replacement following HPV16/18 vaccination - Population-based serological analysis of trial established impact of different vaccination strategies

Penelope Gray[1], Hanna Artemchuk[2], Helena Faust[2], Heljä-Marja Surcel[3,4], Matti Lehtinen[1,2]
[1]Faculty of Social Sciences, Univ. of Tampere; Finland [2]Dept. of Lab Medicine, Karolinska Institute, Stockholm, Sweden; [3]Borealis Biobank and [4]ESIS-EEIG, Oulu, Finland

Summary

Evaluation of human papillomavirus (HPV) type-replacement following different HPV vaccination strategies is done as an anonymous, population-based registry-study in the Finnish Maternity Cohort comparing community-wise over-time (pre- and post-vaccination) HPV seroprevalence rates of randomized, HPV vaccinated (girls-only and gender-neutral) and unvaccinated trial communities.

Background

High risk (hr) Human papillomavirus is a necessary cause of cervical cancer, which is a leading cause of cancer-related morbidity and mortality in women and one of the most common sexually transmitted infections globally. First generation vaccines targeting two the most common hrHPV types (HPV16 and 18) have been licensed for use ten years ago and implemented in national vaccination programmes in high income countries across the world (EMA,2007; FDA, 2006). These vaccines have been shown to provide high vaccine efficacy against vaccine types, cross-protection (against HPV31, 33 and 45) (Brown et al, 2009; Wheeler et al 2012) and additional herd effects against non-vaccine types HPV31, 33 and 35 (Lehtinen et al, 2017). However, concern has been raised about the possibility of HPV type replacement post-vaccination as the vaccine only targets two out of the IARC classified thirteen high risk carcinogenic types (HPV16, 18, 31, 33, 35, 39, 45, 51, 52, 56, 58, 59 and 68) (IARC, 2012; Lehtinen & Paavonen, 2004).

Previous classical vaccination campaigns have been majorly successful without the occurrence of vaccine evolution or adverse changes in pathogen distribution. However, such vaccines have largely targeted childhood illnesses, have conferred lifelong, sterilizing, strain-transcending immunity similar to that conferred by natural immunity, and have targeted pathogens whose survival strategy is dependent on infecting susceptible individual (i.e. children) (Mackinnon & Read, 2007). This is not the case in HPV vaccination; first generation HPV vaccines only target two hrHPV types, the vaccines target different epitopes to that targeted by antibodies produced during the natural humoral immune response to infection, vaccination induces better immunity than naturally acquired immunity (Stanley, 2007), HPV vaccination is targeting a sexually transmitted virus with a differing most at-risk demographic (Garnett & Bowden, 2000) and HPV vaccination does not provide sterilising immunity but is a prophylactic vaccine (Stanley, 2007). Therefore, it would be complacent to assume that the HPV vaccine should have success without adverse consequences to pathogen distribution and or evolution on the similar scale to that of classical vaccines, such as those targeting smallpox, measles and polio.

According to Gause's law, when two species occupy and compete for the same ecological niche, when one species is removed the other will fill the newly vacated ecological niche, in a phenomenon known as type replacement (Gause, 1934). In 2004, Lehtinen and Paavonen expressed concern regarding the possibility of HPV type replacement post-HPV-vaccination; whether competition occurs between vaccine-targeted HPV types and non-vaccine covered types to a degree which could lead to type replacement once the vaccine targeted types have vacated their ecological niche due to vaccination. Type replacement was first observed in practice after the implementation of the *Streptococcus pneumoniae* vaccination programs caused an increase in non-vaccine types, including penicillin resistant non-vaccine types (Weinberger et al, 2011). This principle of competitive exclusion could occur via differing mechanisms (McLean, 1995). If in natural infection a vaccine type confers cross-

reactive immunity against another non-vaccine HPV type, and the vaccine does not confer cross-protection to the same degree as in natural infection, then once vaccination is implemented, it could take away this selective pressure once conferred to the non-vaccine type, resulting in an increase in the non-vaccine type (McLean, 1995). Alternatively, if in the absence of vaccination a vaccine-targeted HPV type can superinfect a person already infected with non-vaccine type, resulting in a reduction or clearance of the non-vaccine type, then once vaccination is implemented removing this limiting pressure on the non-vaccine type, the non-vaccine type may increase (Nowak & May, 1994; McLean 1995). Additionally, in the case of HPV, the low prevalence of HPV types may be indicative that the lack of transmission possibilities may play a greater selective pressure on the distribution types in the general population, however, in the demographic of the population with high risk sexual behaviours, which tend to be assortative, this limiting pressure may be less, which may allow for the effects of competitive exclusion to play a greater pressure in these groups of individuals, which would suggest that type replacement would be more likely in this demographic (Garnett, 2005).

So far there has been conflicting evidence as to whether type replacement does occur post-HPV-vaccination. Several studies of HPV ecology have not found evidence to suggest that vaccine-targeted types compete with non-vaccine types, (which would have been indicative of type replacement) (Tota et al., 2016). A study by Palmroth et al (2010), also found no evidence that naturally acquired immunity provides cross-protection against other HPV types. However, there has been some evidence to suggest the occurrence of superinfection exclusion; in cases of HPV coinfection, vaccine targeted HPV16 has been found to have a blocking effect on other HPV types ability to bind to the extracellular matrix (Biryukov, 2016).

Studies comparing pre- to post-HPV-vaccination non-vaccine HPV rates have also found conflicting results. In Finland, Gray et al. (2017) found no consistent increases in non-vaccine targeted hrHPV types after 4 years of community randomised trial follow-up, although some significant increases in HPV51 and 39 prevalence. Additionally, a recent meta-analysis by Mesher et al. (2016), which encompassed 8 different studies, found some increases in hrHPV 39 and 52, and in possibly carcinogenic (p) HPV types 53 and 73, post-vaccination. However, many of these studies included in the meta-analysis faced problems with bias caused by diagnostic artifact due to the type of PCR methods used and selection bias.

In Finland, the decision was made to launch a community randomised HPV vaccination trial of the bivalent vaccine and different vaccination strategies in 2007 (Lehtinen et al., 2015). The data from the follow-up of this trial provides an excellent resource to evaluate whether type replacement occurs post-vaccination, and the likelihood of it occurring according to different vaccination strategies, with possible linkage to the Finnish Maternity Cohort allowing for the thorough evaluation of type replacement in the non-HPV-vaccinated. The evaluation of HPV type replacement occurrence post-HPV16/18-vaccination is essential in the determination of the future role HPV vaccination programmes, both in Finland and globally.

Aims

The aim of this study is to evaluate whether HPV type-replacement by non-vaccine specific HPV types occurs post-HPV16/18-vaccination in unvaccinated females. Specifically to compare HPV type specific seroprevalence (cumulative incidence) among unvaccinated females under 23 years of age in the thirty-three Finnish communities which participated in the Finnish HPV vaccine community-randomised trial, by vaccination strategy, and pre-vaccination and post-vaccination periods.

Materials & Methods

Community-randomised trial

The randomized community trial comprised 33 Finnish communities, divided into 3 arms: Arm A, Arm B and Arm C (11 communities each) (figure 1). A total of 80,272 individuals born between 1992-1995 were initially identified from the Finnish Population Register. Each arm was assigned a different vaccination strategy with Arm C acting as the control arm. In arm A, the vaccination was gender neutral, with 90% of the participants randomly chosen to receive the HPV vaccine, Cervarix, and 10% to receive the HBV vaccine Engerix. In arm B, only girls were vaccinated with the HPV vaccine, with 90% of females randomly chosen to receive Cervarix and the remaining 10% of females and all males received Engerix. In arm C, all participants received Engerix. The vaccination status was blinded until age 18.5 years for all arm A participants and for all female participants in arm B (Lehtinen et al., 2015). The study visits to administer the vaccine doses took place in the junior high schools.

Finnish Maternity Cohort

The data was obtained by the linkage of the registry of HPV vaccinated individuals with the Finnish maternity cohort (FMC). The FMC serum bank consists of prenatal serum samples from over 96% of all pregnant mothers in Finland, collected since 1983.A total of 8022, first pregnancy/first trimester samples collected during the pre-(2005-2007) and post-vaccination (2008-2010, 2011-2013 and 2014-2016) periods, from women under the age of 23, were from the 33 communities participating in the randomised community trial (table1).

The first time period (2005-2007) is representative of the epidemic state of HPV infections prior to any use of the HPV vaccines, and the following 3 time periods represent the post-implementation era, with gradually increasing vaccination coverage.

Finnish Medical Birth Register

Information on self-reported maternal smoking status shall be gathered by linkage with the Finnish Medial Birth Register via the study subjects' Finnish personal identification number. Self-reported maternal smoking shall be used an indicator variable for behavioural risk taking.

Laboratory Analysis

The serum samples from the Finnish maternity cohort are being analysed using a pseudovirion Luminex serology method, which utilises ELISA with a high-throughput method using multiplexing and a Luminex analyser in order to simultaneously measure for the antibodies to multiple HPV types (HPV6, 11, 16, 18, 31, 33, 35, 39, 45, 51, 52, 56, 58, 59, 66, 68 and 73) and herpes simplex virus type 2 (HSV-2) present in the serum in response to past and present infection at the time of sample donation (Faust et al, 2010; Faust et al, 2013). This shall be done at the Karolinska Institute in Stockholm, Sweden. Samples from HPV vaccinated individuals are identified as outliers due to 100-fold higher HPV16/18 antibody levels and shall be excluded from further analyses.

Statistical Analysis

Over-time trends of type specific cumulative incidence (seroprevalence) (up to 23-years of age) shall be calculated from data collected from the serological analysis of the 7022 serum samples from unvaccinated women under the age of 23 at the time of sample donation to the FMC. Type specific adjusted cumulative incidence ratio (IR) (95% confidence intervals) shall be stratified by arm comparisons: Arm A vs Arm C and Arm B vs Arm C). The HPV specific adjusted odds ratio (OR) (95% confidence intervals) of coinfection with vaccine types HPV16 or HPV18/45 shall be estimated

in each study arm. E-values shall be calculated for both cumulative incidence ratio approach and odds ratio approach, in accordance with VanderWeele and Ding (2017). The adjusted odds ratio estimates and their corresponding confidence intervals shall then be transformed onto the natural log scales. The difference in log odds ratios (95% confidence intervals) between the log (OR) in Arms A and C, and between the log (OR) in Arm B and C shall be estimated. Ratio of odds ratios (95% confidence intervals), comparing Arms A to C, and Arms B to C shall be calculated as according to Altman and Bland (2003).

IRS and ORs shall be adjusted for HSV-2 (as a surrogate of sexual risk-taking behaviour) and maternal smoking status, (as an indicator of general risk taking behaviour), to take account for possible differences in exposure occurrence in communities not due to vaccination and time. Additionally, changes in the ranked seroprevalence of HPV types in the unvaccinated women under the age of 23 years old in the intervention Arms A and B when compared to the control Arm C, shall analysed using Spearman's rank correlation coefficient for the four consecutive time periods.

The statistical analyses shall be conducted using R statistical software version 3.4.3 with Epi package (version 2.15, The R Foundation; https://www.r-project.org/) and ggplot2 package (version 2.2.1, The R Foundation; https://www.r-project.org/), for the graphical presentation of results.

Ethics

The community randomized study obtained permissions from the Ethical Review Board of Pirkanmaa Hospital District (R07113M 14.6.2007) and the Ethical Review Board of North Bothnia Hospital District (EETTMK:111/2009). This population-based serological registry-study within the Finnish Maternity Cohort is being evaluated by the North Bothnia Hospital District's Borealis Biobank review board. Informed consent to use the serum samples from the Finnish Maternity Cohort for research purposes is granted by the pregnant women when they donate their 1st trimester sample for screening of congenital infections.

References

Altman D, and Bland JM (2003) Interaction revisited: the difference between two estimates. BMJ 326: 219.

Biryukov, J. (2016) Superinfection exclusion and competition between multiple high-risk human papillomavirus types during a co-infection. Pennsylvania: Pennsylvania State University.

Brown, D., Kjaer, S., Sigurdsson, K., Iversen, O., Hernandez-Avila, M., Wheeler, C., et al.(2009) The impact of quadrivalent human papillomavirus (HPV; types 6, 11, 16, and 18) L1 virus-like particle vaccine on infection and disease due to oncogenic nonvaccine HPV types in generally HPV-naive women aged 16-26 years. Journal of Infectious Diseases, 199:926–935.

EMA. (2007). Cervarix: EPAR- scientific discussion; Url: http://www.ema.europa.eu/docs/en_GB/document_library/EPAR_-_Scientific_Discussion/human/000721/WC500024636.pdf [accessed September 5th 2017]

Food and Drug Administration, FDA (2006). Approval letter- human Papillomavirus quadrivalent (Types 6, 11, 16, 18) Vaccine, Recombinant. Url: http://www.fda.gov/BiologicsBloodVaccines/Vaccines/ApprovedProducts/ucm111283.htm [accessed September 5th 2017].

Faust, H., Jelen, M., Poljak, M., Klavs, I., Učakar, V., and Dillner, J. (2013) Serum antibodies to human papillomavirus (HPV) pseudovirions correlate with natural infection for 13 genital HPV types. Journal of Clinical Virology, 56(4):336-41.

Faust, H., Knekt, P., Forslund, O., and Dillner, J. (2010) Validation of multiplexed human papillomavirus serology using pseudovirions bound to heparin-coated beads. The Journal of General Virology, 91(7):1840-8.

Garnett, G.P. (2005) Role of herd immunity in determining the effect of vaccines against sexually transmitted disease. Journal of Infectious Diseases, 191 (Supplement 1): S97-106.

Garnett, G.P. and Bowden, F.J. (2000) Epidemiology and control and curable sexually transmitted diseases: opportunities and problems. Sexually Transmitted Diseases, 27(10): 588-99.

Gause, G. (1934). The struggle for existence. Human papillomavirus type replacement following vaccination. Baltimore, The Williams &Wilkins company.

Gray, P., Palmroth, J., Luostarinen, T., Apter, D., Dubin, G., Garnett, G., et al. (2017) Evaluation of HPV type-replacement in unvaccinated and vaccinated adolescent females-Results of a community randomised clinical trial (II). International Journal of Cancer, Submitted.

IARC working group on the evaluation of carcinogenic risk to humans. (2012) Biological agents. IARC Monographs on the Evaluation of Carcinogenic Risks to Humans, 100B: 255-313.

Lehtinen, M., Apter, D., Baussano, I., Eriksson, T., Natunen, K., Paavonen, J., et al. (2015). Characteristics of a cluster-randomized phase IV human papillomavirus vaccination effectiveness trial. Vaccine, 33(10), 1284-1290.

Lehtinen, M., Luostarinen, T., Vänskä, S., Söderlund-Strand, A., Eriksson, T., Natunen´, K., et al. (2017). Gender neutral provides improved control of human papillomavirus types 18/31/33/35 through herd immunity: Results of a community randomized trial (III). Int J Cancer, Submitted.

Lehtinen, M., and Paavonen, J. (2004). Vaccination against human papillomaviruses shows great promise. Lancet, 364(9447): 1731-1732.

Mesher, D., Soldan, K., Lehtinen, M., Beddows, S., Brisson, M., Brotherton, J. M., . . . Thomas, S. L. (2016). Population-level effects of human papillomavirus vaccination programs on infections with nonvaccine genotypes. Emerging Infectious Diseases, 22(10), 1732-1740.

McLean, M. (1995) Vaccination, evolution and changes in the efficacy of vaccines: a theoretical framework. Proceedings of the Royal Society Biological Sciences, 261:389–393.

Nowak, M., and May, R. (1994) Superinfection and the evolution of parasite virulence. Proceedings. Biological Sciences, 255(1342):81-9.

Palmroth, J., Merikukka, M., Paavonen, J., Apter, D., Eriksson, T., Natunen, K., . . . Lehtinen, M. (2012). Occurrence of vaccine and non-vaccine human papillomavirus types in adolescent Finnish females 4 years post-vaccination. International Journal of Cancer, 131(12), 2832-2838.

Palmroth, J., Namujju, P., Simen-Kapeu, A., Kataja, V., Surcel, HM., Tuppurainen, M., et al. (2010) Natural seroconversion to high-risk human papillomaviruses (hrHPVs) is not protective against related HPV genotypes. Scandinavian Journal of Infectious Diseases, 42(5):379-84.

Read, A., and Mackinnon, M. (2007). Pathogen evolution in a vaccinated world. In Evolution in Health and Disease. Oxford University Press.

Stanley, M. (2007). Prophylactic HPV vaccines. Journal of Clinical Pathology, 60(9): 961–965.

Tota, J. E., Ramanakumar, A. V., Villa, L. L., Richardson, H., Burchell, A. N., Coutlee, F., and Franco, E. L. (2016). Cervical infection with vaccine-associated human papillomavirus (HPV) genotypes as a predictor of acquisition and clearance of other HPV infections. The Journal of Infectious Diseases, 214(5), 676-684.

Wheeler, CM., Castellsagué, X., Garland, SM., Szarewski, A., Paavonen, J., Naud, P., et al. (2012) Cross-protective efficacy of HPV-16/18 AS04-adjuvanted vaccine against cervical infection and precancer caused by non-vaccine oncogenic HPV types: 4-year end-of-study analysis of the randomised, double-blind PATRICIA trial. Lancet Oncology, 13:100-110.

Weinberger, D. M., Malley, R., and Lipsitch, M. (2011). Serotype replacement in disease after pneumococcal vaccination. Lancet, 378(9807): 1962-1973.

VanderWeele T, and Ding P (2017) Sensitivity analysis in observational research: introducing the E-value. Annals of Internal Medicine 167 (4): 268-274.

Figure 1: Finnish community randomised trial design.

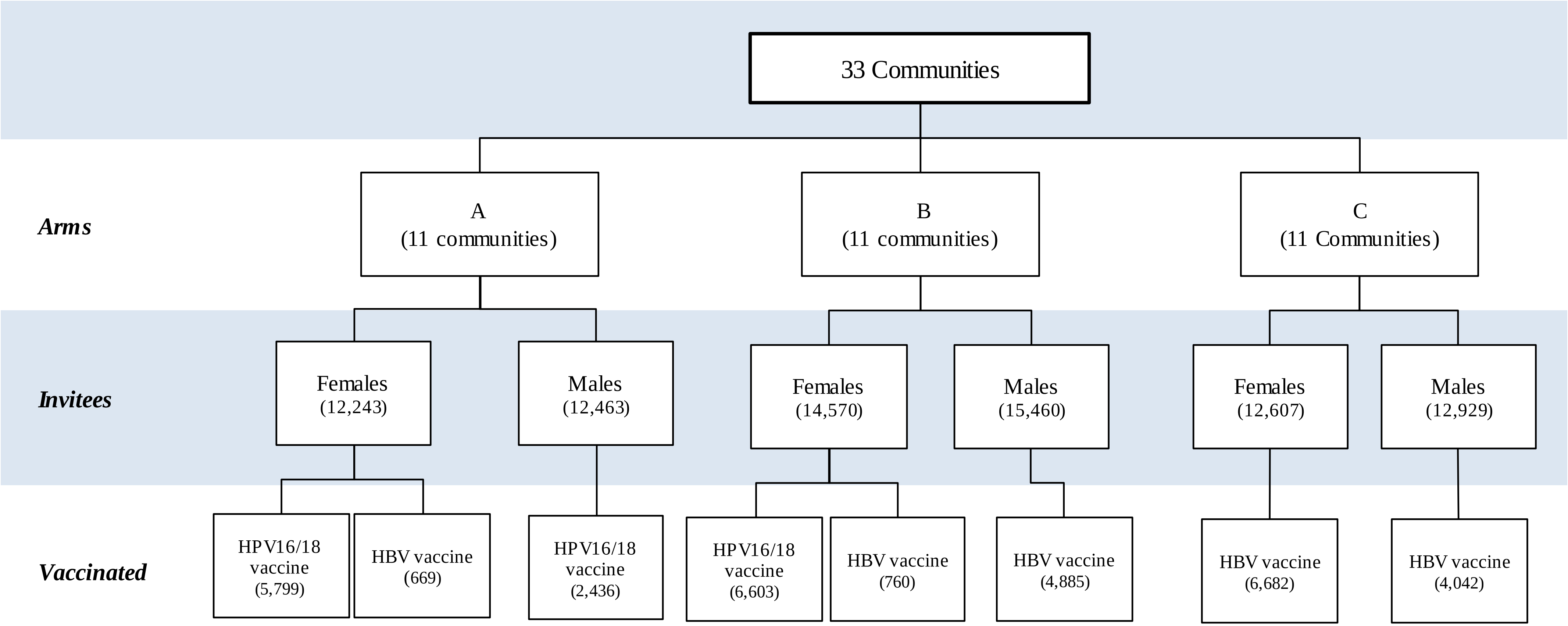

Table 1: Description of serum samples collected from unvaccinated women <23 years of age, from the 33 Finnish communities of the Finnish randomised community trial.

		Time period of sample donation			
Arm	Communities	2005-2007	2008-2010	2011-2013	2014-2016
A	11	11 x 60	11 x 60	11 x 60	11 x 60
B	11	11 x 60	11 x 60	11 x 60	11 x 60
C	11	11 x 60	11 x 60	11 x 60	11 x 60
Total=	33	1980	1980	1980	1980
		5940 (5388*)			1980 (1634*)
				Combined total no. of samples	7920 (7022*)

*Actual number of samples

Table 2: HPV type specific cumulative incidence ratio (95% confidence intervals) (E-Values) among unvaccinated women <23 years of age, from the 33 Finnish communities of the Finnish randomised community trial, comparing intervention Arms A and B to control Arm C.

	Cumulative Incidence Ratio (95% Confidence Intervals)							
	Arm A vs C				Arm B vs C			
HPV type	2005-2007	2008-2010	2011-2013	2014-2016	2005-2007	2008-2010	2011-2013	2014-2016
6								
11								
16								
18								
31								
33								
35								
39								
45								
52								
56								
58								
59								
68								
73								

Table 3: Odds ratio (95% confidence intervals) (E-Values) of a) HPV16 or b) HPV18/45 coinfection with nonvaccine protected HPV types, difference between log(odds ratio) (dOR) (95% confidence intervals) (E-Values) of HPV16 or HPV18/45 coinfection with nonvaccine protected HPV types comparing intervention Arms A/B with control Arm C, and ratio of odds ratio (ROR) (95% confidence intervals) comparing intervention Arms A/B with control Arm C.

	Odds Ratio (95% Confidence Intervals)											
	Arm A				Arm B				Arm C			
**HPV type**	2005-07	2008-10	2011-13	2014-16	2005-07	2008-10	2011-13	2014-16	2005-07	2008-10	2011-13	2014-16
a) **HPV16** (neg) as a reference group												
b) HPV18/45 (neg) as a reference group												

	Difference between log (Odds Ratio) (dOR) (95% confidence intervals)								Ratio of Odds Ratio (ROR) (95% confidence intervals)							
	A vs C				B vs C				A vs C				B vs C			
**HPV type**	2005-07	2008-10	2011-13	2014-16	2005-07	2008-10	2011-13	2014-16	2005-07	2008-10	2011-13	2014-16	2005-07	2008-10	2011-13	2014-16
a) **HPV16** (neg) as a reference group																
b) **HPV18/45** (neg) as a reference group																

Table 4: Changes in ranked distribution of HPV types over four time periods ([I] 2005-2007, [II] 2008-2010, [III] 2011-2013, [IV] 2014-2016) comparing intervention arms to control arm. a) r_s^{I}= (95% confidence intervals), r_s^{II}= ( ),r_s^{III}= ( ), r_s^{IV}= ( ); b) r_s^{I}= ( ), r_s^{II}= ( ),r_s^{III}= ( ) , r_s^{IV}= ( ).

a) Arm A compared to Arm C

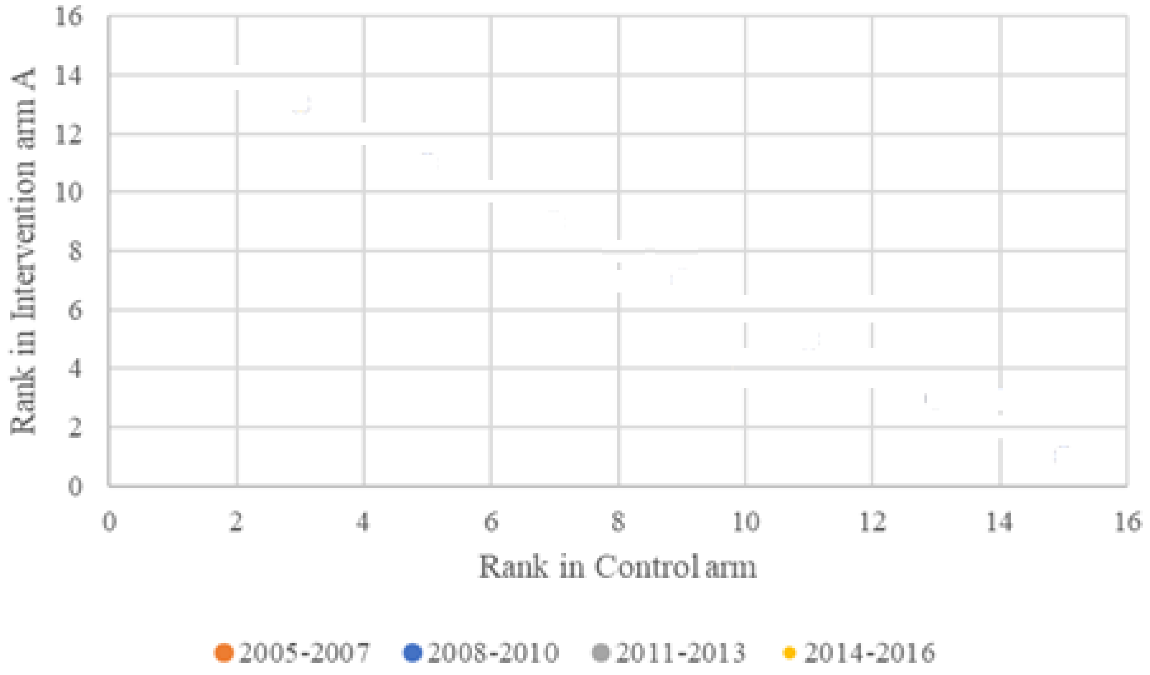

b) Arm B compared to Arm C

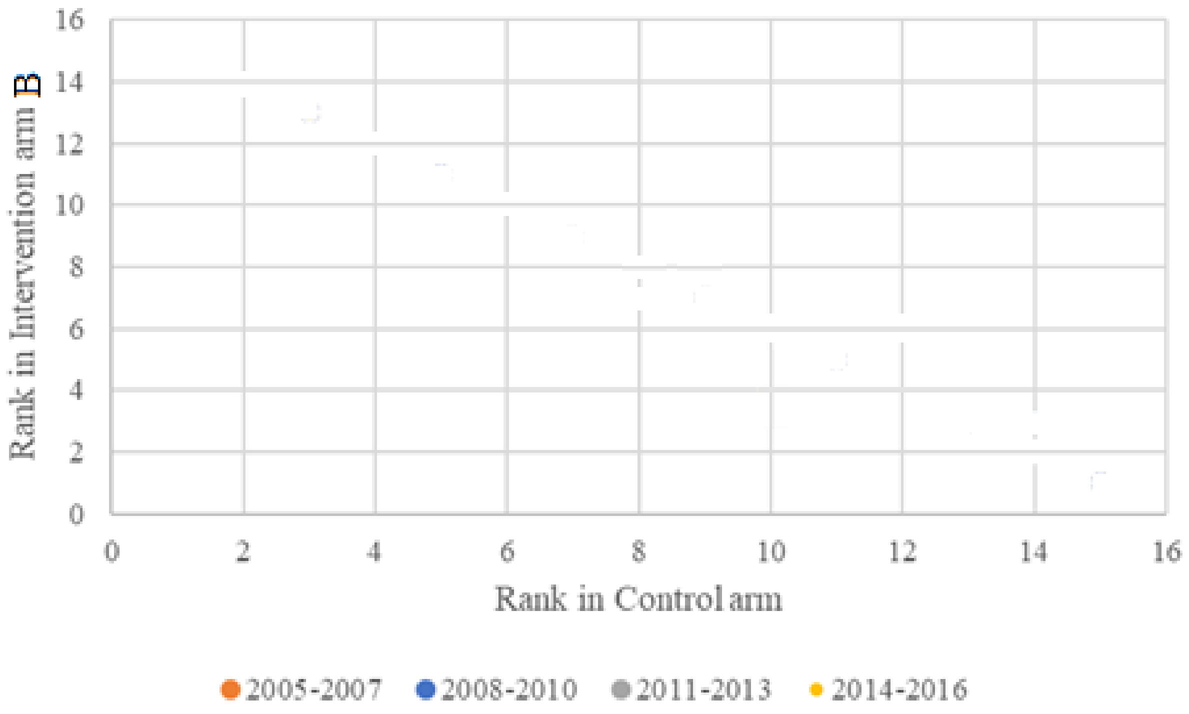

Supplement: S2 Text — (PDF) [file pmed.1003588.s012.pdf]
