# Supplementary material for: Human papillomavirus seroprevalence in pregnant women following gender-neutral and girls-only vaccination programs in Finland: A cross-sectional cohort analysis following a cluster randomized trial
Source: PLoS Med. 2021 Jun 7;18(6):e1003588. doi: 10.1371/journal.pmed.1003588 (PMC8216524; doi:10.1371/journal.pmed.1003588)
Supplement: S3 Text — (PDF) [file pmed.1003588.s013.pdf]

PROMOTING SEXUAL HEALTH IN POPULATION-BASED COHORTS OF THE YOUNG

Matti Lehtinen[1,2], Dan Apter[3], Kari Hemminki[4], Eero Pukkala[5], Heljä-Marja Surcel[1], Jorma Paavonen[1,6] [1]Natl Public Health Institute; [2]Univ of Tampere; [3]Family Federation of Finland; [4]Deutches Krebsforschungszentrum, DKFZ; [5]Finnish Cancer Registry; [6]Univ of Helsinki, Finland

CONTENTS

- BACKGROUND 1
- DESIGN OF INTERVENTIONS 2-3
- INTERVENTIONS 4-5
- FOLLOW-UP 5-8
- EFFECTS ON DISEASE OCCURRENCE 8-9
- IMPACT 9
- REFERENCES 10

SUMMARY **Rationale** High risk (hr) human papillomaviruses (HPV) are the major cause of human cancer, with cervical cancer and other HPV-associated cancers estimated to cause >5% of all cancers. On the other hand, *Chlamydia trachomatis* infection is asssocitaed with at least half of infertility cases. Increasing incidences of HPV-associated anogenital cancers and infertility in many EU countries are due to decreasing age at sexual debut, increasing risk taking sexual behaviour, and increasing occurrence of hrHPV and *C. trachomatis* infections. However, highly efficacious population level hrHPV vaccination and *C. trachomatis* screening will get started next year. The following considers: How to implement the interventions, What are their effects on molecular epidemiology of HPV & *C. trachomatis* and How the interventions are reliably monitored?

Objectives Population based adolescent cohorts are used for longitudinal intervention studies on HPV and *C. trachoma* infections, with a particular reference to using newly established cohorts and biobanks to evaluate the implementation and long-term effectiveness of the interventions and to generate new knowledge on disease etiology translatable to health-promotion strategies.

Benefits Proof of principle on how population-based cohorts and biobanks can be used to rapidly and cost-efficiently generate new knowledge on implementation and evaluation of major preventive programmes, and improved understanding of the molecular epidemiology of major diseases, their causes, and eradication of both the causes and the disease.

Work content Vaccination of population-based cohorts of adolescents (ages 13-15 yrs) with 70% coverage using HPV or hepatitis B virus (HBV) vaccines in a community randomized setting with three arms: A) HPV girls/HPV boys, B) HPV girls/HBV boys, C) HBV girls&boys in 7 communities/ arm (1,500-4,350 early adolescent/community), yields 80% power for the demonstration of 85% difference of hrHPV prevalence between A and C, assuming sexual contacts in the same community, vaccine efficacy of 95%, expected hrHPV prevalence of 20% in unvaccinated 18 yr-olds and 15% population mixing. The cohorts are followed with repeated HPV and *C. trachomatis* samplings and host genotypings exploiting existing biobank systems. Need for boostering and rescreening is also determined in this context. Molecular epidemiology of breakthrough infections/type-replacement of HPVs, and genetic epidemiology of the infected vaccinees and recurrent *C. trachomatis* infections are studied. Combinations of screening for *C. trachomatis*, hrHPV infections and cervical cancer, as well as prospects for sexual health edication will be evaluated. Health economics of the intervention will be assessed from the molecular epidemiology point of view. The community randomized setting to investigate relevance of the intervention in developing countries is fully exploited.

Impact The only effective way of controlling common STDs is inclusion of new preventive measures into national vaccination/screening programmes in a coordinated and evaluable manner.

(Protocol supported by the Finnish Cancer Society grant between 2007-2009)

PROMOTING SEXUAL HEALTH IN POPULATION-BASED COHORTS OF THE YOUNG

M Lehtinen, D Apter, K Hemminki, E Pukkala, H-M Surcel, J Paavonen Natl Public Health Inst; Univ of Tampere; Family Federation of Finland; DKFZ; Finnish Cancer Registry; Univ of Helsinki

BACKGROUND Occurrence of common STDs such as *Chlamydia trachomatis* and human papillomavirus (HPV) infections has steadily increased in young Nordic women and men (af Geijerstamm et al., 1998, Hiltunen-Back et al. 2003, Wilson et al. 2002, Laukkanen et al. 2003). High-risk (hr) HPVs and *C. trachomatis* now have prevalences of 30% and up to 5% among 20-year old women (Auvinen et al. 2005). Increasing incidences of cervical cancer (CxCa) are also found (Bray et al. 2005). Prediction for CxCa incidence in 2002 for 30-39 yr-old Finnish women was 2.2./100.000 (Engeland et al. 1993) but the observed incidence was 6.1. Increased risk taking sexual behaviour is the probable explanation of both phenomena (Laukkanen et al. 2003).

Fortunately, HPV vaccines based on virus-like particles (VLPs) are very effective in the prevention of HPV infection with vaccine efficacies (VE) of >90% (Harper et al. 2004, Villa et al. 2005). VE of preventive vaccines against infections with HPV16/18, and against the precursors of cervical carcinoma (high grade cervical intraepithelial neoplasia, CIN2+) is being tested in two large phase III trials. Finland is participating both trials with population-based enrolment and long-term follow-up of 3,500 16-18-year old HPV vaccinees, 3,500 "placebo" vaccinees, and 15,000 18-19-year old non-vaccinees. VE against cervical carcinoma we will know by 2012 (Lehtinen et al. 2006a,b).

The registration of personal identification numbers of individuals belonging to one of the three cohorts enables follow-up by different population based health registers. The follow-up includes ongoing biobanking, and for example monitoring of HPV antibody levels from blood samples donated over time by the vaccinees to the Finnish Maternity Cohorts. The existing infrastructure can be used to assess effectiveness of HPV vaccination and *C. trachomatis* screening.

Significant efforts have been devoted to building models that predict the cost-effectiveness of HPV vaccination and *C. trachomatis* screening programs (Paavonen et al. 1998, Honey et al. 2002, Hu et al. 2004, Sanders and Taira 2003, Stamm 2004, Taira et al. 2004). There is an agreement that both HPV vaccination and *C. trachomatis* screening of adolescents can be cost effective when compared to other public health interventions. Whether HPV vaccination can be cost saving is now studied by considering herd immunity in the models. The numerous assumptions in the proposed setting can now be validated. Our strategy is to show effectiveness of population-based HPV vaccination and *C. trachomatis* screening from evaluable implementation of the interventions as community randomized trials (Brookmeyer&Chen 1998, Campbell et al. 2004, Lehtinen and Paavonen 2003).

Vital questions on HPV and *C. trachomatis* epidemics and prevention of associated diseases can be pursued by conducting large scale studies based on longitudinally followed population cohorts and biobanks like the ones described herein: 1) Cross-protection between HPV types, 2) The microbial and host determinants of the immune protection, 3) Duration of protection after vaccination or screening, and 4) Determinants of progression from infection to cancer or to infertility.

It is difficult to predict if vaccination interferes with the natural spectrum of different HPVs, i.e., whether the epidemiology of infections with non-vaccine HPV types will be changed by vaccination/eradication of a limited number of HPV types. Interference between genomes of particular HPV types has been observed (McLaughlin-Drubin and Meyers 2004). To further clarify this important issue we will analyze the incidence and persistence of all types of genital papillomaviruses within the communities that have received effective HPV16/18-vaccine or not. In addition, we will investigate the viral gene expression profiles to learn about the interdependence between individual HPV types within their natural and vaccination modified habitats.

Cervical cancer is known to have a strong hereditary component (Magnuson et al. 1999, Hemminki et al. 1998, 2001). Several studies have implicated certain HLA class II alleles in determining whether HPV16-exposed women will have a persistent infection or not (Lehtinen and Paavonen 2001), which in turn may be a key determinant of cervical cancer risk. However, virtually nothing is known about whether genetic factors will determine immune response following HPV vaccination.

DESIGN OF INTERVENTIONS **Mathematical models** of STDs have concentrated on patterns of sexual risk taking behaviour to focus on heterogeneity in risk and the pattern of mixing between different sexual activity groups within the population (Gregson et al. 2002). Less focus has been on the natural history of the infection and the disease. This is an issue for HPV and *C. trachomatis* infections where severe sequelae occur in a fraction of the population a long-time after infection. Thus, modelling will use standard frameworks for capturing patterns of behaviour and concentrate on developing a valid description of disease pathogenesis in a model for spread of the infections. So far, calculations have been made exploring the impact of different HPV types on cervical cancer but modelling the types separately (Hughes et al. 2002). This, however, fails to capture the potential ecological interaction of the organisms. We will explore the interactions between the two micro-organisms and between two HPV types with a range of levels of type-specific immunity.
Mathematical models developed use ordinary and partial differential equations to explore the pattern of HPV and *C. trachomatis* infections and progress to disease as infections age. Age and time progress concomitantly so that partial differential equations with respect to age and time can be used to provide an age-structured model of the spread of the infections. This requires stratification into age and sexual activity groups to estimate some of the parameters controlling incidence of the infections. Parameters can be estimated using such a framework by maximum likelihood methods to compare observed and modelled patterns of incidence as the parameters are altered. The statistical validity of such an approach depends upon an adequate model structure and exploration of the impact of other parameters which will be explored. Validity has also been explored by reference to over-time patterns of HPV infections and cancer incidence (Barnabas et al. 2006). The model developed will provide a framework within which to explore the **objectives:**

1. HPV and *C. trachomatis* seroprevalence data in combination with sexual behaviour data is used for the maximum log-likelihood estimation of transmission probabilities (β). The framework will be used to incidence predictions associated with behavioural and biological changes.
2. To consider the role of changes in HPV incidence and the age of infection associated with changes in the number of sexual partners, mixing matrix and age at sexual debut, and distribution of HPVs and *C. trachomatis* with different levels of persistence in increased disease incidence.
3. Models will be validated based on goodness of fit between predicted&observed cancer incidences
4. To model HPV and *C. trachomatis* infections, progression to cervical cancer and infertility, and the effect of interventions: vaccination and different versions of organised screening programs.

Simulation studies Following infection a number of different paths can be followed through the possible compartments, each representing a postulated stage of disease pathogenesis. The first three compartments are asymptomatic HPV-infection, LSIL (mild dysplasia) and HSIL (moderate to severe dysplasia). Regression of infection is assumed to result in lifelong immunity to HPV type 16. The fourth compartment represents cervical cancer (CC). Cancer survivors (CS) form the last compartment. Ten percent of asymptomatic HPV infections, that progress directly to HSIL, rather than through LSIL, representing rapidly progressive infection. Men can either be susceptible, infected with HPV or protected by vaccination. Each stage is assumed to regress, persist or progress according to a transition matrix amended from Myers and McCrory (2000). Screening is assumed to prevent 80% of cervical cancers. A simulation study on HPV will:
1. Model national demographics, including estimated sexual behaviour patterns.
2. Consider assortative mixing by sexual activity class and age group.
3. Choose transmission probability (β) according to age-specific HPV incidences & run model.
4. Compare model output with observed HPV seroprevalence by sexual activity class and age.
5. Calculate and compare log-likelihood for each β using the observed and model prevalences.
6. Consider the effect of sexual behaviour changes: using the chosen β look at the effects of changing contact rate, mixing matrix and age at sexual debut on cervical cancer incidence.

This work will serve to parameterize and test models of different HPV vaccination policies to identify the most effective and cost-effective policies. The comparison and integration of observational studies with models of progress to disease and models of the transmission dynamics of the HPVs will allow a number of areas to be explored. These include impact of ecological changes induced by vaccination changing the pattern of HPVs, the targeting of vaccines, and the interaction of vaccination and other programmes to prevent infections/associated cancers.

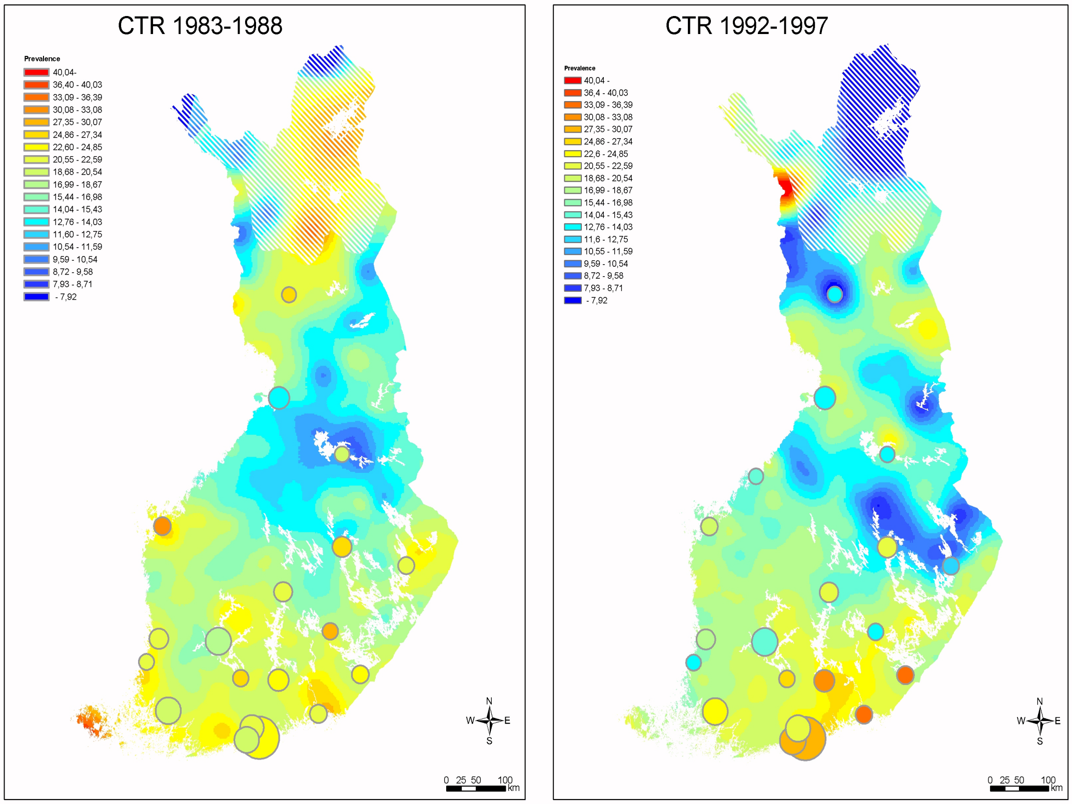

Figure 1. Distribution of *C. trachomatis* (CTR) seroprevalance in females in 1983-88&1992-97

Stratification of communities Community wise STD seroprevalences will be assessed by a versatile multiplex serology platform recently developed in one of our laboratories that permits analyzing sera for the presence of antibodies to a variety of protein antigens. It is based on bacterial expression of antigens as glutathione S-transferase (GST) fusion proteins and *in situ* affinity purification *via* glutathione-linked casein on a solid plastic support. Antibodies to different microbes can be quantified by standard enzyme coupled secondary antibodies in color reactions (Sehr et al. 2002, Lehtinen et al. 2003).

All (>96%) pregnant women in Finland have donated first trimester sera for screening of congenital infections, and since 1983 the serum samples have been stored, and forms the Finnish Maternity Cohort (Anttila et al. 2001). Random subsamples of 8,000 young pregnant women are being used for determination of prevalence trends of common STDs (Lehtinen et al. 2006c). In addition sera from the two ongoing phase III HPV vaccination trials involving 7,000 initially 16-18 -yr old girls in 20 vaccination sudy site communities will be used (Lehtinen et al. 2006a,b). Population of the 20 communities with vaccination study sites (Helsinki and Tampere excluded) varies between 100,000 and 35,000. By community the size of one birth cohort in the target ages (13 to 15 yrs) varies between 1,450 and 500. The study site communities will be randomized by taking into account the over-time trends of common STDs (HPV and *C. trachomatis*) seroprevalences (Figure 1).

INTERVENTIONS **Target communities** Vaccination will take place at secondary schools in the target communities by personnel of study sites existing after the phase III hrHPV vaccination trials. There are currently 20 such study sites. The study subjects will be early adolescents between 13 to 15 years of age in the study communities. Coverage of national vaccination programme in the two countries is 93%, and $\geq$70% of the birth-cohort probably participate in the intervention.
Personal identifiers together with study numbers and pertinent vaccination data will be registered into the Registry of Vaccinees at the National Public Health Institute according to the permission of the data protection agency. Linkage of the Registry of Vaccinees with the population-based Finnish Maternity Cohort will be used both for active and long-lasting monitoring of the antibody levels.

Design and randomization for vaccination Sample size calculations for the community randomized intervention (considering both direct VE and herd immunity) to show significant decrease in the prevalence of hrHPV due to direct VE and herd immunity are based on Brookmeyer & Chen (1998), where protective efficacy:

$$PE = \frac{T(n;K,2)\#\ SD}{\sqrt{(n)}\ CI_{unvac}}$$

Vaccination takes place before sexual debut at the age 13 to 15 yrs with hrHPV vaccines (individually randomized 30 to 70%/community) or with a beneficial hepatitis B-virus (HBV) vaccine to allow the separate evaluation of effectiveness stemming from direct VE and herd immunity. The randomization is by communities stratified for over-time STD seroprevalence with the following assumptions: >50% of the hrHPV infections are acquired within 4 years from sex debut (Collins et al. 2002) from 3-year older or younger partners having sexual contacts in the same community with a population mixing of 15%, VE of 95%, and expected 20% prevalence of hrHPV in unvaccinated 18-19 year olds, when crossover vaccination takes place. To assess the effectiveness hrHPV prevalence comparisons: A) HPV girls&HPV boys, B) HPV girls/HBV boys, vs. C) HBV girls&boys are made. For A vs. C 7 communities (1,500-4,350 vaccinees/community) per arm yields 80% power for demonstrating 85% reduction in HPV case prevalence (Figure 2).

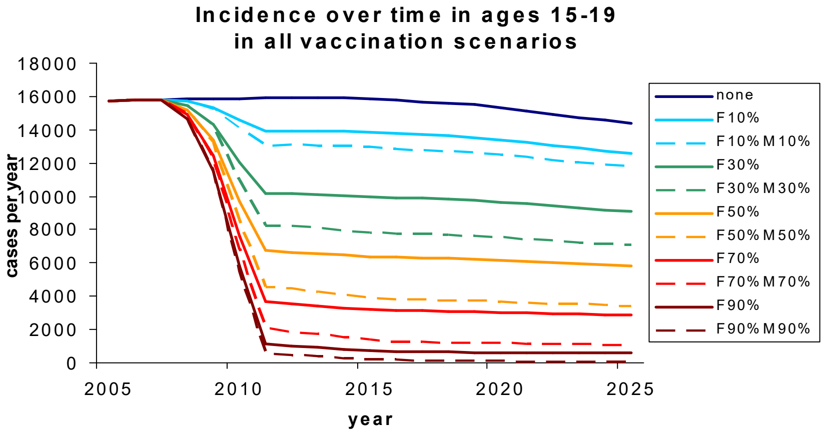

Figure 2. Eradication of HPV16 cases assuming continuing vaccination and lifelong immunity.

Vaccines and crossover vaccination Multivalent HPV virus-like-particle vaccines manufactured by one of the two vaccine manufacturers (Glaxo Smith Kline or Merck) will be used immediately after licensure. Aim for this study is to use HPV type combination in a multivalent vaccine tailored according to the epidemiological situation in the target areas to cover ≥80% of the population attributable fraction of hrHPVs in cervical cancer. There is increasing evidence for cross-protection of the HPV16/18 vaccine(s) towards related hrHPV types (Dubin et al. 2005), which enables achievement of this goal already next year. As a beneficial vaccine against another STD hepatitis B virus (HBV) vaccine(s) will be used. Cross-over vaccination at the age of 18-19 with hrHPV or HBV vaccines, and blood and cytological sampling will be at the study sites.

Design for hrHPV&*C. trachomatis* screening First void urine (FVU) samples will be used for *C. trachomatis* PCR testing. In the ongoing phase III HPV vaccination trials the testing is repeated annually. This has made it possible to determine the community-wise *C.trachomatis* incidences and rates of recurrent infections in the cohorts of 22,000 16- to19-year old young women.
From 2006/7 hrHPV and *C.trachomatis* prevalence among the 18- to 19-yr old vaccinees will be assessed by cervico-vaginal and FVU home sampling of the intervention communities. The use of such homesampling and *C.trachomatis* data protected delivery of results through a safe web page (www.rokotiitus.net) is expected to yield a high compliance among the adolescents. Visits for organized gynaecological sampling will be organised for a random sample (10%) of the vaccinees at the study sites. The home-sampling and organized sampling visits will start in 2006/7 for the unvaccinated birth cohorts born in 1988, -89, -90, etc. to get the baseline results at the beginning of the community randomized interventions.
The effectiveness of hrHPV vaccination among 18- to 19-year old young adults will be evaluated in the community randomized setting. The reduction in hrHPV prevalence at 18- to19-yrs of age will be compared to hrHPV prevalence of communities in the un-vaccinated arm. The long-term outcomes of interest (CIN3+ incidence) will be analysed based on the registries and Finnish Cancer Registry.
The effectiveness of annual *C. trachomatis* screening of 18- to 19-year old young adults will be evaluated in a community randomized setting. In one arm of the study site communities young adults will be screened (and treated if found positive for *C. trachomatis*) two times at the ages of 18- and 19- years. The reduction in *C. trachomatis* prevalence at 19-yrs of age will be compared to *C. trachomatis* prevalence of communities in the un-screened arm. The long-term outcomes of interest (recurrent infection, gynaecologic morbidity) will be analysed based on the registries and other selected registries of the National Public Health Institute, (e.g. Infectious Disease Registry), STAKES (e.g. Hospital Discharge Registry) and Finnish Cancer Registry.

FOLLOW UP **Persistent infections** with HPV16 or HPV18 confer a higher risk for developing high-grade CIN when compared to infections by other hrHPV types (Schlecht et al., 2001). Although infections caused by the low risk (lr) HPV and hrHPV types seem to cluster (Thomas et al. 2000) there is competition between the different HPV types at the level of carcinogenesis (Luostarinen et al. 1999, 2004). Following pneumococci vaccination new types emerge to replace the ecological niches created by removing the vaccine types (Lipstich 1997, Byington et al. 2005). Whether both HPV type categories change after reduction of hrHPVs remains to be seen.

Active follow-up The aim of the active follow-up is to determine the causes (viral or host factors) of changes in the prevalence of vaccine and non-vaccine HPV types following vaccination. Special emphasis will be put on breakthrough HPV infections and type-replacement by new hrHPV types, and need for boostering and vaccination of susceptible individuals against new HPV types.

This trial gives the opportunity to define immunocorrelates for protection: the titer of neutralizing antibodies that confer protection and the titer at which there will be breakthrough infections (as in the hepatitis B context) using newly developed pseudovirion neutralization assays (Pastrana et al. 2004). This is crucial to identify individuals that might require booster vaccination and to establish general booster vaccination schedules.

We will take advantage of the blood and cytological samples taken during the crossover vaccination of 18-19 year olds to investigate whether the reduction of HPV16 and HPV18 infections has influenced the appearance and gene expression profile of the vaccine HPV types or other HPV types. We will also analyse the genetic factors that explain breakthroughs or type-replacements.

Passive follow-up Females will be invited to cytological screening for cervical cancer from the ages of 25, respectively. The screening results and DNA extracted from the cytological ThinPrep samples can be retrieved by record linkage of the Registry of Vaccinees with the Finnish Cancer Registry. Finnish Cancer Registry is maintained by law and receive personal identification number (PIN) based notifications of virtually all cases of cancer and cervical intraepithelial neoplasia grade III (98%) that are histologically confirmed. The neoplasia data and diagnostic biopsies can be retrieved by record linkage.

Finnish Maternity Cohort (FMC) and Infectious Disease Registry at the National Public Health Institute are maintained by law. They collect and store 1st trimester serum samples of >95% of pregnant women and all Finnish citizens, respectively. Each year 55,000 serum samples and 30,000 new females enter the FMC, and >50% of the vaccinated womens enter the FMC by the age of 30. The Infectious Disease Register collects data, including personal identifiers, from newly diagnosed sexually transmitted infections. Notifications of *C. trachomatis* are made both by clinicians and laboratories, and notifications of hrHPVs will be included.

Hospital discharge registry at the Research&Development Centre for Social Welfare&Health is maintained by law and collects data on hospitalisations with pertinent ICD diagnoses.

Virological analyses Typing of HPV by DNA hybridization procedures, such as Reverse Line Blot (RLB) assay, is sensitive and well-validated. However, the application of these assays towards high-throughput analyses is limited. Here, we describe the development of a quantitative and sensitive high-throughput procedure for the identification of multiple high- and low-risk genital HPV genotypes in a single reaction. Multiplex human papillomavirus genotyping (MPG) is based on the amplification of HPV DNA by a general primer PCR (GP5+/GP6+) and the subsequent detection of the products with type-specific oligonucleotide probes coupled to fluorescence-labelled polystyrene beads (Luminex suspension array technology). Up to 100 different HPV types can be detected simultaneously with MPG and the method is fast and labour-saving.

The Luminex system described above for the serology has been adapted to multiplex nucleic acid hybridizations with biotinylated PCR products (Dunbar et al. 2003; Ye et al. 2001). For HPV DNA typing we use general primer PCRs to generate amplimers of 65 or 150 bp nucleotides from different hrHPV types (Jacobs et al. 1997; Kleter et al. 1999). The amplimers are then denatured and hybridized onto HPV type-specific single-stranded 17-22 bp oligonucleotides chemically linked to the individual and subsequently pooled bead species. The amplimer DNA strand of the polarity opposite to the coupled oligonucleotide contains a biotin-labeled primer that, in case of a positive hybridization, is detected through a streptavidin-phycoerythrin on a particular species of beads that in turn are identified by their internal color as tag for the HPV type.

By the MPG all 22 HPV types examined (6, 11, 16, 18, 31, 33, 35, 39, 42, 43, 45, 51, 52, 56, 58, 59, 66, 68, 70, 73 and 82) were detected with high specificity and reproducibility (median inter-plate coefficient of variation <10%). Detection limits for the different HPV types varied between 100 and 800 pg of PCR products. We compared the performance of MPG to an established RLB assay on GP5+/GP6+ -PCR products (Kleter et al. 1999, van den Brule et al. 2002) derived from 94 clinical

samples. The evaluation showed an excellent agreement (kappa = 0.922) but also indicated a higher sensitivity of MPG. MPG appears to be highly suitable for large-scale epidemiological studies and vaccination trials as well as for routine diagnostic purposes (Schmitt et al. submitted).
For the active follow up extension of Luminex-based MPG to all 14 available high-risk types (HPV16, 18, 31, 33, 35, 39, 45, 51, 52, 56, 58, 59, 66, 68) and 12 low-risk types (HPV6, 11, 32, 34, 40, 42-44, 53, 54, 70, 74) and validation the assay against the results of a large series of HPV-typed cervical specimens available through collaborations with Christine Clavel, Reims and Thomas Iftner, Tübingen will be made. Following the established strategy we will define various experimental parameters (size of the PCR amplimer and its position within the HPV genome and conditions of hybridization) to obtain maximal sensitivity (about 10 copies per assay, i.e. 1000 copies/cervical specimen) and specificity (differentiation between the HPV types).
The next step will be development of a Luminex-based RT PCR reverse hybridization assay to analyze the expression profile of individual HPV types. For generation of transcript-specific amplimers from total RNA by RT PCR we will develop type-specific primers for all hrHPV and the most relevant lrHPV types, specific for each of the ORFs E6, E4 and L1. Expression of these genes correlates to early, delayed and late stages of infection and indicates a virus replication vs. viral persistence/malignant progression of the infected epithelium (Middleton et al. 2003). To exclude positive results due to contaminating DNA within the RNA preparation we will, whenever possible, use pairs of primers that discriminate between spliced and genomic sequences. The system will be developed stepwise by first analyzing RNA from cells transfected *in vitro* with HPV expression plasmids, from cervical cancer cell lines and HPV-positive raft cultures before we proceed to clinical specimens. The detection system will be in accordance to the HPV genotyping system (see above) using type- and gene-specific oligonucleotides for coupling to the Luminex beads.
Analysis of samples from the vaccination trial will be the final step in the active follow-up. We will use our Luminex-based MPG detection system to perform genotyping from 2,000 samples. After identification of the HPVs present within a specimen we will perform RNA profiling analyses from a subset of specimens from breakthrough HPV infections and wild-type HPVs (n = 500).

Genetic analyses of the infected hosts Familial risk of cervical cancer has been established in studies utilizing the Swedish family registers (Hemminki et al. 1999, 2003, Magnusson et al. 1999). The high hereditability estimates for cervical neoplasms (0.22 to 0.34) may be explained by uncontrolled socioeconomic and behavioural variables, such as split families and risk-taking behaviour (Pukkala, Thesis 1995), known HLA associations of persistent HPV infection and cervical cancer (Lehtinen and Paavonen 2001) by yet unknown genetic factors, or by any combinations of these. Since persistent HPV infection is a prerequisite of cervical cancer it is highly probable that genetic factors predispose individuals to the development of a persistent HPV infection. Furthermore, it is conceivable that genetic factors also play a role in susceptibility to/acquisition of HPV infections (breakthroughs or infections with non-vaccine hrHPVs).
We will evaluate the impact of host factors on the risk of acquiring breakthrough HPV infections and on the replacement of the vaccine HPV types in vaccinated individuals. The studies will be carried out on years 3 to 5 of the project when virological control data of the vaccinated subjects become available. The community randomized trial setting generates ample herd immunity that allows the evaluation of both these issues (Lipstich M, personal communication). The study will include all subjects with a breakthrough infection (estimated n = 300), and all subjects with a type-replacement (estimated n = 600). As controls we use a random subsample of subjects who have been successfully immunized (n = 500), and those who have received HBV vaccination (n = 500) to give a baseline profile of infection. All the four groups will be compared to each other. The results will be given as odds ratios (ORs) associating a defined genetic polymorphism with breakthrough infection and/or type-replacement. The statistical power will be over 90% to detect an OR of 1.5 for a gene with an allele frequency of 0.2 with a sample size 600 vs. 500 ($\alpha = 0.05$).

The analysis of single nucleotide polymorphism (SNPs) of putative candidate genes in the four groups described above (total n=1900) will be tentatively carried out using Luminex technology (seee WP1). For HLA-typing standard PCR techniques are already available. In addition to the HLA-typing, we plan to analyse 200 candidate genes per year adding up 600 genes for the three years of study, totalling 1.14 million genotypes. Whole-genome approaches currently available, including Illumina 100k array and Affymetric 500k SNP chip are expensive (about 500 Euros each, 1900 x 500 = 0.95 millio Euros) and have at present not been prioritised. However, the final choice of the technology to be exploited will be made during year 2 of the project.
Among the genes of interest HLA-haplotypes have been associated with persistent HPV infection and cervical cancer, and they will be tested. Among other genes are those related to innate immunity and apoptosis (such as Toll-like receptor, death receptors, CASP8, RB and p53), and immune and cytokine pathways (e.g. interleukins, TNF-alpha, interferons). The genes will be chosen as likely susceptibility factors based on the available literature on host response to viral infections. The genotyping will be done at DKFZ who have ample experience with many of the above genes in breast and other cancers by using mainly the Taqman technology (Frank et al. 2006).

EFFECT ON DISEASE OCCURRENCE **Background** Although the necessary roles of hrHPV and *C.trachomatis* infections in cervical cancer etiology and infertility are established, the fact that prevention against hrHPVs and *C.trachomatis* are available is paradoxically necessitating larger and more reliable etiological studies. Knowledge of population-attributable proportions of different subtypes and burden of disease is required for surveillance. Validation of intermediate end-points includes elucidation of their progression determinants: e.g. investigations of whether vaccination also protects the small proportion of HPV-exposed subjects that would have been destined to develop some of the HPV-associated cancers (Mork et al. 2001), and whether prevention of *C. trachomatis* reinfections prevents infertility (Paavonen et al. 1998). The underlying trends in risk factor prevalences will affect disease incidence predictions and estimation of effects of HPV vaccination, of *C. trachomatis* screening and of possible combinations of HPV vaccination and screening.
Longitudinal studies nested in population biobank cohorts enable more reliable and efficient study designs for understanding etiology, as well as evaluating preventive strategies. However, some prerequisites apply: Large cohorts with long enough follow-up and large numbers of prospective cases. It is possible to link biobanks with quality-assured population-based registries to enable population-representative studies with minimal case ascertainment bias. Archival cervical screening smears have been used for molecular epidemiology both for HPV and *C. trachomatis* PCR (Wallin et al. 2003). The smears are stored for at least 5 years, which enables longitudinal studies.
The Finnish Maternity Cohort contains venous blood specimens collected during 1983-2005. The samples have been taken at the Maternity Care Units from almost all (>98%) pregnant women in Finland during early pregnancy (I trimester) for screening of syphilis, hepatitis B and HIV infections. For a one-year cohort of women (60,000 individuals) samples taken at hospitals after delivery are also available. Two or more serum samples are available from about half of the 750,000 women.

A large prospective study on cervical cancer The objectives are to: **1.** Obtain an unbiassed estimate of how cervical cancer-causing exposures interact in the disease etiology (different HPVs, smoking, other STDs, genetic susceptibility), **2.** Investigate if the causative exposures vary between populations by region and/or calendar time, **3.** Expand the data base on which HPVs that are currently found in cervical cancer, **4.** Provide reliable input data for modelling the effect of various HPV vaccination and screening strategies. A sound scientific basis for understanding of etiology and underlying risk factor trends in cervical cancer incidence is a prerequisite for surveillance strategies.

A large prospective study on infertility The objectives are to: **1.** Obtain an unbiassed estimate of how infertility-causing exposures interact in the disease etiology (transient, persistent or recurrent *C. trachomatis* infections, genetic susceptibility), **2.** Investigate if the causative exposures vary between populations by region and/or calendar time, **3.** Provide reliable input data for modelling the effect of various *C.trachomatis* screening strategies. A sound scientific basis for understanding of etiology and underlying risk factor trends in incidence of infertility is a prerequisite for surveillance of such strategies.

The study bases are defined as the women participating in the consortium population biobank cohorts: Registry linkages with the corresponding national cancer registries have identified >750 cases of invasive cervical cancer that have occurred >1 month after blood sampling. Registry linkages with population-based and comprehensive cytology and histopathology registries will determine the exact cervical screening history of each woman (to obtain data on protective effect of screening as well as enable retrieval of archival specimens for molecular testing). Since the study aims to not only determine relative risks, but to estimate exposure prevalences among controls, 5 controls per case will be selected, matched for sex, age at blood sampling (+/-2 years), biobank cohort and time of withdrawal of blood sample (+/- 2 months). All laboratory analyses are performed on blinded samples.
Similar studies on cervical carcinoma in situ (CIS) contain almost 8 times as many prospectively occurring cases. Studies on CIS are of interest to determine at what stage cofactors act, and to validate intermediate end-points. As these studies will be very large they will have suffiecient power to analyses occurrence and trends over time also of rare HPV types.

Laboratory analyses Serology. Type-specific antibodies to HPV types 6, 11, 16, 18, 31, 33 and 73 will be measured by the Luminex method as markers of cumulative exposure to the different HPVs. HPV16 and 18 are causative HPV types included in the vaccines. HPV31 and 33 are causative types not included in the vaccines. HPV6, 11 and 73 may interact with the other HPV types. Although currently only assays for 7 genital HPV types are validated, the Luminex can be expanded to simulataneous analysis of up to 100 antigens and the plan is to to establish and validate assays for all genital HPV types. *Chlamydia trachomatis* antibodies will be measured by ELISA.
Genetic studies. Markers of susceptibility will be measured as decribed above. All cervical specimens are requested from the diagnosing pathology laboratories. The diagnosis is re-evaluated by an expert pathologist. The sections are tested for HPV using a general primer PCR that detects virtually all known genital HPV types, followed by HPV typing by hybridisation with 100 HPV type-specific probes bound to 100 differently colored beads, whereafter the type is determined by flow cytometry in the Luminex system (see above). Negative samples are also tested with a PCR system with very short amplimer length (developed by partner 6), that is very sensitive particularly for samples with partially degraded DNA (Neighbor primer PCR (NP-PCR).

IMPACT Community-randomized implementation of vaccination and screening policies conducted post-licensure can be in the case of preventive HPV vaccination or *C. trachomatis* (hrHPV) screening viewed as a totally new type of implementing promotion of adolescent health. It involves stringent designs for both the implementation and its evaluation. It also links the prevention efforts to ambitious molecular studies on carcinogenesis and microbial pathogenesis**.**
The proposed research is the most accelerated way of translating existing research results on the efficacy of HPV vaccines and on the new screening tools into public health policy. At the same time, it generates a study for improved understanding of molecular events in susceptibility to infections and to their sequelae, and for optimisation and cost-efficient monitoring of programs aiming to control the two most common STDs.

REFERENCES

af Geijerstamm V, Wang Z, Lewensohn-Fuchs I, et al. Int J Cancer 76:341-344, 1998
Anttila T, Saikku P, Koskela P, et al. JAMA 285:46-51, 2001
Auvinen E, Niemi M, Malm C, et al. Scand J Infectious Dis 37:873-876, 2005
Barnabas R, Laukkanen P, Koskela P; et al. PLoS Medicine, in press 2006
Bray F, Loos AH, McCarron P, et al. Cancer Epidemiol Biomakers Prev 14:677-686, 2005
Brookmeyer R, Chen Y-Q. Stat Med 17:2122-2132, 1998
Byington CL, Samore MH, Stoddard GJ, et al. Clin Infect Dis 41:21-29, 2005
Campbell MK, Elbourne DR, Altman DG. BMJ 328:702-708, 2004
Collins S, Mazloomzadeh S, Winter H, et al. Br J Obstet Gynecol 109:442-446, 2002
Dubin G. 21st International Human Papillomavirus Conference, Voncouver 2005
Dunbar SA, Vander Zee CA, Oliver KG, et al. J Microbiol Methods 53: 245-252, 2003
Frank B, Hemminki K, Shanmugam KS, et al. Carcinogenesis, in press 2006
Gregson S, Nyamukapa CA, Garnett GP, et al. Lancet 359:1896-1903, 2002
Harper DM, Franco EL, Wheler CM, et al. Lancet 364:1757-1765, 2004
Hemminki K, Dong C, Vaittinen P Int J Cancer 82:775-781, 1999
Hemminki K, Li X. Int J Cancer 103:105-109, 2003
Hiltunen-Back E, Haikala O, Kautiainen H, et al. Sex Transm Dis 30:737-741, 2003
Honey E, Augood C, Templeton A, et al. Sex Transm Infect 78:406-412, 2002
Hu DM, Hook EW III, Goldie SJ. Ann Intern Med 141:501-513, 2004
Jacobs MV, Snijders PJ, van der Brule AJ, et al. J Clin Microbiol 35:2508-2517, 1997
Kleter B, van Doorn LJ, Schrauwen L, et al. J Clin Microbiol 37:2508-2517, 1999
Laukkanen P, Koskela P, Pukkala E, et al. J Gen Virol 84:2105-2109, 2003
Lehtinen M, Dillner J. Sex Transm Infect 78:4-6, 2002
Lehtinen M, Paavonen J. Int J STD&AIDS 12:771-776, 2001
Lehtinen M, Paavonen J. Int J STD&AIDS 14:787-792, 2003
Lehtinen M, Pawlita M, Zumbach K, et al. Am J Obstetr Gynecol 188:49-55, 2003
Lehtinen M, Paavonen J. Lancet 364:1737-1738, 2004
Lehtinen M, Idänpään-Heikkilä I, Lunnas T, et al. Int J STD & AIDS, 17:1-10, 2006a
Lehtinen M, Herrero R, Mayaud P, et al. Vaccine, in press 2006
Lehtinen M, Kaasila M, Pasanen K, et al. Int J Cancer, in press 2006c
Lipsitch M. PNAS 94:6571-6576, 1997
Luostarinen T, Lehtinen M, Bjorge T, et al. Eur J Cancer 40:1058-1067, 2004
Magnusson P, Sparen P, Gyllenstein U, et al. Nature 400:29-30, 1999
McLaughlin-Drubin ME, Meyers C. Virology 321:173-180, 2001
Middleton K, Peh W, Southern S, et al. J Virol 77:10186-10201, 2003
Mork J, Lie S-K, Glattre O, et al. N Engl J Med 344:1125-1131, 2001
Myers ER, McCrory DC, Nanda K, et al. Am J Epidemiol 151:1158-1171, 2000
Paavonen J, Puolakkainen M, Paukku M, Sintonen H. Obstet Gyneocol 92:292-298, 1998
Pastrana DV, Buck CB, Pang YY, et al. Virology 321:205-216, 2004
Sanders GD, Taira AV. Emerg Infect Dis 9:37-48, 2003
Schlecht NF, Kulaga S, Robitaille J, et al. JAMA 286:3106-3114, 2001
Sehr P, Muller M, Hopfl R, et al. J Virol Methods 106:61-70, 2002
Stanberry LR, Spruance SL, Cunningham AL, et al. New Engl J Med 347:1652-1661, 2002
Stamm W. Ann Int Med 141:570-572, 2004
Taira AV, Neukermans T, Sanders GD. Emerg Infect Dis 10:1915-1923, 2004
van den Brule AJ, Pol R; Fransen-Daalmeijer N, et al. J Clin Microbiol 40:779-787, 2002
Villa L, Costa R, Tetta C, et al. Lancet Oncology 6:271-278, 2005
Wallin K-L, Wiklund F, Luostarinen T, et al. Int J Cancer 101:371-374, 2002
Ye F, Li MS, Taylor JD, et al. Hum Muta 17:305-316, 2001

Analysis Plan for effectiveness analysis of phase IV trial HPV-040
RAP – report analysis plan

Overview/Contents – kept as similar as possible to template from Toufik so that the effectiveness sections can be added to the main RAP easily.

1. list of amendments to the RAP
2. introduction
3. objectives
4. study design overview
5. conduct of study
6. data evaluation: criteria for evaluation of objectives
    a. endpoints
    b. study cohorts
    c. derived and transformed data
    d. group description
    e. interim analysis
    f. demography
    g. effectiveness analysis
    h. methodology for computing CI
7. any change from protocol
8. individual listings
9. annex 1: template of tables

List of abbreviations

ATP	According-to-protocol
CI	Confidence interval
GEE	Generalised estimating equations
ITT	Intention-to-treat
RAP	Report analysis plan
SAS	Statistical software SAS 9.1.3

1. List of amendments to the RAP

2. Introduction

This document summarises the study features as per protocol (sections 3-5) and the planned statistical analyses (section 6). A layout of the tables as they will be produced in the statistical report is available in annex 1.

3. Objectives (effectiveness)

Primary

- To demonstrate the overall (direct and indirect) effectiveness of GSK Biologicals' HPV-16/18 vaccine in reducing the prevalence of HPV-16/18 genital infection in females approximately 18.5 years of age, following community-based vaccination of 12 - 15 year old females only (Arm B versus Arm C).
- To demonstrate the overall (direct and indirect) effectiveness of GSK Biologicals' HPV-16/18 vaccine in reducing the prevalence of HPV-16/18 genital infection in females approximately 18.5 years of age, following community-based vaccination of 12 - 15 year old females and males (Arm A versus Arm C).

Secondary

- To compare the overall (direct and indirect) effectiveness of GSK Biologicals' HPV-16/18 vaccine in reducing the prevalence of HPV-16/18 genital infection in females approximately 18.5 years of age following community-based HPV vaccination of 12 - 15 year old females versus HPV vaccination of 12 - 15 year old females and males (Arm B versus Arm A).
- To evaluate the overall (direct and indirect) effectiveness of GSK Biologicals' HPV-16/18 vaccine in reducing the prevalence of genital infection with oncogenic HPV types (overall and individually, including but not necessarily limited to HPV-16, -18, -31, -33, -35, -45, -52 and -58) in females approximately 18.5 years of age, following community-based vaccination of 12 - 15 year old females only (Arm B versus Arm C).
- To evaluate the overall (direct and indirect) effectiveness of GSK Biologicals' HPV-16/18 vaccine in reducing the prevalence of genital infection with oncogenica HPV types (overall and individually, including but not necessarily limited to HPV-16, -18, -31, -33, -35, -45, -52 and -58) in females approximately 18.5 years of age, following community-based vaccination of 12 - 15 year old females and males (Arm A versus Arm C)…

Tertiary…

- To evaluate the indirect effectiveness of GSK Biologicals' HPV-16/18 vaccine in reducing the prevalence of HPV-16/18 genital infection in females approximately of 18.5 years of age receiving control vaccine, following community-based vaccination of 12-15 year old females and males (Arm A versus Arm C), and community-based vaccination of 12 - 15 year old females only (Arm B versus Arm C). This objective will explore the potential for the vaccine to induce herd immunity effect.
- To evaluate the direct effectiveness of GSK Biologicals' HPV-16/18 vaccine in reducing the prevalence of HPV-16/18 genital infection in females approximately of 18.5 years of age receiving HPV vaccine following community-based vaccination of 12 - 15 year old females and males (Arm A versus Arm C), and community-based vaccination of 12 - 15 year old females only (Arm B versus Arm C).
- To describe the frequencies of genital infections with individual oncogenic HPV types other than HPV-16 and HPV-18 in females approximately 18.5 years of age following vaccination of 12-15 year old females and males (Arm A, Arm B and Arm C).
- To evaluate the occurrence of CIN3+ lesions and invasive cervical cancer as well as other cancers associated with HPV…

4. Study design overview
From protocol

5. Conduct of study
From protocol

6. Data evaluation for effectiveness

Summary (as in protocol)

The analysis of effectiveness will be an intention-to-treat analysis, so will include all communities that have been enrolled in the trial. The statistical analysis of effectiveness will be done by comparing the HPV-16/18 PCR prevalence rates in communities in different trial arms: A vs. C and B vs. C (co-primary endpoints), and in trial arms A vs. B (first secondary endpoint). The statistical analysis of the second and third secondary endpoints will be done by evaluating the difference in HPV-16/18/31/45/52/58/33 PCR prevalence rates in communities in different trial arms A vs. C and B vs. C. The statistical analysis of the tertiary endpoints will be done by determining the difference in HPV-16/18 prevalence in the study participants not vaccinated with HPV-16/18 vaccine in the different community arms A vs. C and B vs. C. The unit of analysis for these analyses of effectiveness is the community.

Testing of the null hypotheses of no difference in prevalence between the trial arms for each endpoint will be done using the Mantel-Haenszel one degree of freedom chi-square statistic adjusted for clustering (ref). The effectiveness will be further explored using generalised estimating equations (GEE) (ref) and further analysis will include adjustment for covariates. Examples of variables that will be considered include, but are not necessarily limited to, community size, behavioural and adherence characteristics, HPV vaccine coverage, HPV opportunistic vaccination and HPV prevalence at baseline. With 11 communities per trial arm the study will be at the borderline of the minimum number of communities required for the validity of GEE. Therefore a random effects logistic normal model will also be used to check the sensitivity of the GEE conclusions.

6.1 endpoints

Primary

- HPV-16 and/or HPV-18 DNA positivity (by PCR) in all approximately 18.5-year old female study participants.

Secondary

- HPV-16 and/or HPV-18 DNA positivity (by PCR) in approximately 18.5-year old female study participants in Arms A and B.
- Oncogenic HPV DNA positivity (by PCR) (overall and individually) in all approximately 18.5-year old female study participants.

Tertiary

- HPV-16/18 DNA positivity (by PCR) in approximately 18.5-year old female study participants in Arms A, B and C.

- Oncogenic HPV DNA positivity (by PCR) in approximately 18.5-year old female study participants in Arms A, B, and C.
- Confirmed CIN3+ lesions identified for oncogenic HPV types (e.g. HPV-16, -18, -31, -33, -35, -45, -52 and -58).

6.2 study cohorts used in effectiveness analysis

Total cohort
The total cohort will include all study participants from all communities for whom HPV DNA PCR data will be collected at the time of effectiveness evaluation phase. The total study cohort will include all communities of the trial.

Total HPV Vaccinated cohort (nb need to decide whether to include all participants with at least one vaccine administration documented and if so whether to perform the analysis per treatment actually administered?) the analysis of effectiveness is an intention-to-treat analysis so I think we would do one analysis for all people regardless of number of vaccinations received.
The total HPV vaccinated cohort will include all HPV vaccinated study participants for whom HPV DNA PCR data will be collected at the time of effectiveness evaluation phase. This cohort will include all communities of the trial.

Total Control Vaccinated cohort (again need decision on inclusion and analysis)
The total control vaccinated cohort will include all control vaccinated (with HBV) study participants for whom HPV DNA PCR data will be collected at the time of effectiveness evaluation phase. This cohort will include all communities of the trial.

6.3 derived and transformed data

Number positive in a community
Number positive in the total cohort, within each arm and within each stratum
Number positive in the total HPV vaccinated cohort, within each arm and within each stratum
Number positive in the total control vaccinated cohort, within each arm and within each stratum
(and number with cytological abnormalities and CIN2+ lesions associated with high risk HPV types)
Number participants in a community
Number participants in the total cohort, within each arm and within each stratum
Number participants in the total HPV vaccinated cohort, within each arm and within each stratum
Number participants in the total control vaccinated cohort, within each arm and within each stratum
Prevalence in a community
Prevalence in the total cohort, within each arm and within each stratum
Prevalence in the total HPV vaccinated cohort, within each arm and within each stratum
Prevalence in the total control vaccinated cohort, within each arm and within each stratum
Coefficient of variation/intracluster correlation coefficient for each stratum
HPV vaccination coverage in each community
HPV vaccination coverage in the total cohort, within each arm
HPV vaccination coverage in the total HPV vaccinated cohort, within each arm
HPV vaccination coverage in the total control vaccinated cohort, within each arm

Vaccine effectiveness – for each of the endpoints
Indirect effectiveness – for each of the endpoints?
Size of communities
Adherence characteristics (ie one dose, two doses, three doses etc)
Behavioural characteristics
HPV opportunistic vaccination
HPV prevalence at baseline

6.4 group description
Not sure what this is

6.5 interim analysis

As planned in the protocol an interim analysis for the primary objectives will be triggered once visit 5 HPV DNA results from all female residents born in 1992 and 1993 are available. No stopping rules are associated with this analysis as this interim analysis is not powered for the secondary objectives.

The purpose of this analysis is to gain early experience on the effectiveness of HPV vaccination… The interim analysis of the primary objectives will be done as per the full effectiveness analysis of the primary objectives and an adjusted alpha will be used. For the primary endpoint, the overall alpha of 0.05 will be split into 0.021 for the interim analysis and 0.039 for the final analysis.

6.6 demography

Tables generated:

Table # in ref of annex 1	Abbreviated title	Final analysis	Interim analysis
	Number of subjects in each cohort?		
	Summary of demographic characteristics (total cohort)		
	Summary of demographic characteristics (total vaccinated cohort)		
	Summary of demographic characteristics (total control vaccinated cohort)		

Note whether in the report or as a supplementary table/figure

6.7 effectiveness analysis

6.7.1 Calculate coefficient of variation/intracluster correlation coefficient.
Data required:
- size of each cluster in each arm,
- prevalence in each cluster in each arm,
- overall prevalence in each arm,
- total number clusters in the study,
- total number of individuals in the study,
- and calculate the pooled mean square errors between and within clusters.

The intracluster correlation coefficient (ρ) is calculated as described in Donner and Klar () for each stratum and then an overall estimate is determined as the average over the strata. For each stratum;

$$\hat{\rho} = \frac{MSC - MSW}{MSC + (m_0 - 1)MSW}$$

where MSC and MSW are the pooled mean square errors between and within clusters respectively.

$$MSC = \sum_{i=1}^{2}\sum_{j=1}^{k_i} m_{ij}\left(\hat{P}_{ij} - \hat{P}_i\right)^2 / (K-2)$$

$$MSW = \sum_{i=1}^{2}\sum_{j=1}^{k_i} m_{ij}\hat{P}_{ij}\left(1 - \hat{P}_{ij}\right) / (M-K)$$

$$m_0 = \left[M - \sum_{i=1}^{2}\overline{m}_{Ai}\right] / (K-2)$$

where i is the trial arm, j is the cluster, and mij is the size of the jth cluster in arm i. Pij is the prevalence within each cluster j in each group i. K is the total number of clusters in the study and M is the total number of individuals in the study.

The calculation of the intracluster correlation coefficient is entirely manually coded (ie not using pre-written code/functions) in SAS.

6.7.2 Calculate adjusted Mantel-Haenszel chi square statistic to examine null hypotheses.

The adjusted Mantel-Haenszel chi square statistic will be used to examine each of the null hypotheses.

Data Required:

- intracluster correlation coefficient,
- size of each cluster,
- total number of subjects,
- number of cases in each arm in each stratum,
- number of people in each arm in each stratum.

Null hypotheses (first two only for interim analysis) (description in brackets is the HPV vaccination strategy):

1. Prevalence of HPV16/18 in Arm B (females vaccinated only) = Prevalence of HPV16/18 in Arm C (control)
2. Prevalence of HPV16/18 in Arm A (females and males vaccinated) = Prevalence of HPV16/18 in Arm C (control)
3. Prevalence of HPV16/18 in Arm A (females and males vaccinated) = Prevalence of HPV16/18 in Arm B (females vaccinated only)
4. Prevalence of high risk HPV types in Arm B (females vaccinated only) = Prevalence of high risk HPV types in Arm C (control)
5. Prevalence of high risk HPV types in Arm A (females and males vaccinated) = Prevalence of high risk HPV types in Arm C (control)

6. Prevalence of HPV16/18 in control vaccinated cohort in Arm B (females vaccinated only) = Prevalence of HPV16/18 in control vaccinated cohort in Arm C (control)
7. Prevalence of HPV16/18 in control vaccinated cohort in Arm A (males and females vaccinated) = Prevalence of HPV16/18 in control vaccinated cohort in Arm C (control)
8. Prevalence of HPV16/18 in HPV vaccinated cohort in Arm B (females vaccinated only) = Prevalence of HPV16/18 in total HPV vaccinated cohort in Arm C (control)
9. Prevalence of HPV16/18 in HPV vaccinated cohort in Arm A (males and females vaccinated) = Prevalence of HPV16/18 in total HPV vaccinated cohort in Arm C (control)
10. Prevalence of cytological abnormalities and CIN2+ lesions associated with high risk HPV types in Arm B (females vaccinated only) = Prevalence of cytological abnormalities and CIN2+ lesions associated with high risk HPV types in Arm C (control)
11. Prevalence of cytological abnormalities and CIN2+ lesions associated with high risk HPV types in Arm A (females and males vaccinated) = Prevalence of cytological abnormalities and CIN2+ lesions associated with high risk HPV types in Arm C (control)

Example data table for each of the null hypotheses:

Stratum	Intervention	No Clusters	No cases	No subjects
1	Arm 1	n_{11}	A_{11}	M_{11}
	Arm 2	n_{21}	A_{21}	M_{21}
	Total		A_1	M_1
2	Arm 1	n_{12}	A_{12}	M_{12}
	Arm 2	n_{22}	A_{22}	M_{22}
	Total		A_2	M_2
3	Arm 1	n_{13}	A_{13}	M_{13}
	Arm 2	n_{23}	A_{23}	M_{23}
	Total		A_3	M_3

The adjusted Mantel-Haenszel chi square value is calculated as described by Donner and Klar (). Clustering correction factors are calculated for each of the two arms being compared as follows:

$$C_{1j} = \left[\sum_m m\left[1+(m-1)\hat{\rho}\right] n_{1jm} \right] / M_{1j}$$

and

$$C_{2j} = \left[\sum_m m\left[1+(m-1)\hat{\rho}\right] n_{2jm} \right] / M_{2j}$$

where n_{1jm} and n_{2jm} are the number of experimental and control group clusters in stratum j having exactly m subjects, ρ is the intracluster correlation coefficient, and the summation is taken over all clusters of size *m*. The Mantel-Haenszel chi square value is calculated as:

$$\chi^2_{MHA} = \frac{\left[\sum_{j=1}^{s} \frac{A_{1j}\left(M_{2j}-A_{2j}\right)-A_{2j}\left(M_{1j}-A_{1j}\right)}{M_{1j}C_{2j}+M_{2j}C_{1j}}\right]^2}{\sum_{j=1}^{s} \frac{M_{1j}M_{2j}A_j\left(M_j-A_j\right)}{\left(M_{1j}C_{2j}+M_{2j}C_{1j}-1\right)M_j^2}}$$

where s is the number of stratum. Under the null hypothesis (assumed constant across strata) that the intervention odds ratio is 1, χ^2_{MHA} has an approximate chi-square distribution with one degree of freedom.

The calculation of the Mantel-Haenszel chi square value, including the clustering correction factors and confidence intervals is entirely manually coded (ie not using pre-written code/functions) in SAS.

Also calculate an odds ratio with confidence intervals.

6.7.3 Use a GEE to explore effectiveness

The generalised estimating equations will extend a logistic regression model to allow for clustering.
Normal logistic regression:
$LogOdds_{ij} = \beta x_{ij}$

This extension is done by specifying a correlation matrix that describes the association between different individuals in the same cluster. In a cluster randomised trial it is assumed that correlations between individuals in the same cluster are the same – this is an ‘exchangeable’ correlation matrix. I think this will be an analysis at individual and cluster level to explore the impact of covariates that are at both levels? Or for this do we do the random effects model? So the repeated measure would be cluster?

To define a regression model using GEE, need to define:

- distribution of the dependent variable – binomial
- link function - logit
- independent variables – first and most important is trial arm
- covariance structure of the repeated measurements – exchangeable

The first model will be:
Log odds of HPV = TrialArm
should be individuals with repeated measure being clusters.

Further models will adjust for covariates including community size, behaviour and adherence characteristics, HPV vaccination coverage, age at vaccination, HPV opportunistic vaccination and HPV prevalence at baseline.

The analysis will be done using PROC GENMOD in SAS.

6.7.4 Use a random effects model to test the results of the GEE

Random effects logistic regression includes a random effect that explicitly allows for correlations (clustering).
$LogOdds_{ij} = \beta x_{ij} + \mu_i$
The model will include a random effect μ_i for each cluster.
So looking at clusters within strata – still a cluster level analysis – unless we have individual level characteristics that we'd like to explore so we can extend the model? Not sure about this part.

Initial model would be:
Log odds of HPV = TrialArm + μ_i

Further models will adjust for the same covariates as in the GEE,

The analysis will be done using PROC MIXED in SAS

6.8 methodology for computing CI?

Not sure that this is needed as already mentioned CI for MH

Annex 1

Tables

Table 1

Stratum	Arm	Cluster	No cases	No female	No male	Prevalence	Cluster Size	Prevalence at baseline	Vaccination Coverage
1	1	1							
1	1	2							
1	1	3							
1	1	4							
1	2	5							
1	2	6							
1	2	7							
1	2	8							
1	3	9							
1	3	10							
1	3	11							
1	3	12							
2	1	13							
2	1	14							
2	1	15							
2	2	16							
2	2	17							
2	2	18							
2	3	19							
2	3	20							
2	3	21							
3	1	22							
3	1	23							
3	1	24							
3	1	25							
3	2	26							
3	2	27							
3	2	28							
3	2	29							
3	3	30							
3	3	31							
3	3	32							
3	3	33							

Table 2

Stratum	Arm	Cluster	ID	Cohort	Gender	Age at test	Seropositive	No vacc doses	Oppo Vacc	Age first sex
1	1	1	1							
1	1	1	2							
1	1	1	3							
…	…	…	…							

Table 3

Stratum	Intra-cluster correlation coefficient
1	
2	
3	

Table 4

Hypothesis	Mantel-Haenszel Chi square	P value	Mantel-Haenszel Odds Ratio	Confidence Intervals
1				
2				
3				
…				

Results of GEEs

Results of Random effects models

.
