# Supplementary material for: Human papillomavirus seroprevalence in pregnant women following gender-neutral and girls-only vaccination programs in Finland: A cross-sectional cohort analysis following a cluster randomized trial
Source: PLoS Med. 2021 Jun 7;18(6):e1003588. doi: 10.1371/journal.pmed.1003588 (PMC8216524; doi:10.1371/journal.pmed.1003588)
Supplement: Attachment — Submitted filename: Response to the reviewers.docx [file pmed.1003588.s014.docx]

Dr Clare Stone, PhD

Acting Chief Editor

PLOS Medicine

14^th^ May 2020

Dear Clare Stone,

Thank you very much for your consideration of our previously submitted manuscript (PMEDICINE-D-20-00112R1). We now enclose a revised version of this research article entitled “Gender-neutral vaccination is the key to HPV16 herd effect when vaccination coverage is limited: a cluster-randomised trial of vaccination strategy” for you further consideration for publication in PLOS Medicine.

We would like to thank the editorial board and the reviewers for their time spent reviewing this research article. We have now revised the manuscript in response to the specific points raised in the comments from the reviewers and the editorial board. Below is our point-to point response to these comments.
-----------------------------------------------------------

**Requests from the editors:

*In the title and throughout the paper, the term "community RCT" is used, do the authors mean "cluster RCT" which might be the more commonly recognised term? (in that randomization is of groups of individuals, not individuals themselves). The authors could consider using cluster RCT instead, this might be more generally understood and recognised.**

**Re:** Thank you for raising this point. Indeed “cluster-randomised trial” is probably a more well recognised and understood term. We have therefore now revised the title to read *“Gender-neutral vaccination is the key to HPV16 herd effect when vaccination coverage is limited: a cluster-randomised trial of vaccination strategy”*, and revised the methods similarly.

***Although the authors use CONSORT to aid reporting of the trial, it would be good to explicitly mention this in the methods (and call out there the supporting file which basically constitutes the CONSORT checklist), and also to consider using the specialised CONSORT extension for cluster RCTs (**[**http://www.consort-statement.org/extensions?ContentWidgetId=554**](http://www.consort-statement.org/extensions?ContentWidgetId=554)**) as this contains specific advice for particular elements of design and analysis that should be elaborated in more detail for a cluster RCT.**

**Re:** We have now used the specialised CONSORT extension for cluster randomised trials, included in the submission, and have explicitly mentioned this in the methods as advised (page 9, lines 11-12). ***In the abstract, it would be good to state how many clusters were in each randomized arm in the trial.**

**Re:** We have now amended the abstract to inform the reader that 11 communities were in each randomised arm in the trial.

***In the last sentence of the Abstract Methods and Findings section, it would be good to include a brief description of any key limitation(s) of the study's design/methods.**

**Re:** We have now described a key limitation of this study’s design in the abstract Methods and findings section (page 2, line 20).

***At this stage, we ask that you include a short, non-technical Author Summary of your research to make findings accessible to a wide audience that includes both scientists and non-scientists. The Author Summary should immediately follow the Abstract in your revised manuscript. This text is subject to editorial change and should be distinct from the scientific abstract. Please see our author guidelines for more information:** [**https://journals.plos.org/plosmedicine/s/revising-your-manuscript#loc-author-summary**](https://journals.plos.org/plosmedicine/s/revising-your-manuscript#loc-author-summary)

**Re:** We have now added a short non-technical Author summary of this article which can be found in the submitted revised version immediately after the abstract (page 3).

***If possible, please tweak the in-text referencing style to match the PLOS Medicine format (numerals in square brackets, ie [1, 2]) - if using referencing software this should be fairly straight forward.**

**Re:** We have now tweaked the in-text referencing style as requested to match that of the PLOS Medicine format.

***Per the reviewers' comments, it would be good to explain in more detail in the manuscript and acknowledge the degree of novelty over and above prior studies also examining this question.**

**Re:** We have now revised the introduction to this effect (page 5, lines 20 & 24, page 6, lines 1-2) and were already discussing in detail the novelty of this study in comparison to previous studies in the discussion (page 16, lines 5-11, page 17, lines 1-7).

***In the Methods, it would be good to spell out the randomization procedures more clearly, per the CONSORT statement (either standard or cluster CONSORT should explain what needs to be detailed), but specifically an explanation of how allocation to arms was concealed and so on.**

**Re:** We have explained the randomization procedures, and how the (community-level) allocation to the arms was not concealed on page 8 (lines 1-17).

-----------------------------------------------------------

**Comments from the reviewers:

Reviewer #1: "Gender-neutral vaccination is the key to HPV16 herd effect when vaccination coverage is limited: a community-randomised trial of vaccination strategy" investigates the effects of moderate gender-neutral vs. girls-only vs. control (none) human papillomavirus (HPV), through a community-randomized trial in Finland, on herd immunity. The main analysis involving post- vs. pre-vaccination HPV prevalence/correlation was performed on 8,022 qualifying unvaccinated pregnent women whom were 23 years old or under and resident in the 33 involved communities, between 2005 and 2016. It was concluded that only gender-neutral vaccination results in herd protection against HPV16/18 (and not girls-only or no vaccination), when vaccination coverage is moderate.

The major strengths of this study lie in the original large-scale community-randomized design, and the statistical analysis attempting to take linked attributes (e.g. maternal smoking, HSV-2 seropositivity) into account. The general findings are also in accordance with expectations and modelling attempts (e.g. [12]), given the primary spread of HPV through intercourse.**

We would like to thank the reviewer for acknowledging the unique nature of our study.

**However, there are a number of key concerns:**

**1. Firstly, relatively similar analyses (comparing gender-neutral versus girls-only impact on HPV prevalence) on the same data have been published by some of the same authors (i.e. citations [16],[8]). In fact, some of the details available in these prior work (e.g. on the nature of the 33 communities, with each community having at least 35,000 inhabitants, and being at least 35km from each other) would probably be appropriately included as supplementary material here. In any case, the same broad conclusions had been reached in [16]: "In conclusion, while gender-neutral strategy enhanced the effectiveness of HPV vaccination for cross-protected HPV types with low to moderate coverage, high coverage in males appears to be key to providing a substantial public health benefit also to unvaccinated females".**

Thank you for this comment. We have included information regarding the nature of the 33 communities, the study design and related details in the “Study Design”, subsection of the methods, as is also necessitated by the CONSORT extended checklist for cluster randomised trials (Methods pages 7-9). As discussed in length in the manuscript this study is distinct from the previous follow-up of this community randomised trial, and novel as we now evaluate the different vaccination strategies using a measure of cumulative incidence (HPV-serology), whereas previous follow-up was restricted to one-time HPV DNA positivity as a measure of transient infection.

**The immediate reaction would then be on the additional contributions of this manuscript, on top of and over these previous works ([16],[8]). From what could be understood, the main distinction was that cervicovaginal samples taken at 18.5 years from all (vaccinated/unvaccinated) female participants were used for PCR analysis in [16], as opposed to seroprevalance amongst unvaccinated pregnant females at or under 23 years only - but also from non-participating cohorts - for this study. In general, the authors might consider stressing the presence of their directly-relevant previous work, and describe more precisely the relative strengths/weaknesses of this paper in comparison to those previous works.**

**Re:** We have now revised the manuscript to stress the presence of the previous follow-up of this community-randomised trial. The differences and the comparative strengths and weaknesses of this approach are described and mentioned in the following passages: in the introduction on page 5, lines 20 & 24, page 6, lines 1-2, and in the discussion on page 16, lines 5-11 and page 17, lines 1-7.
 **2. While the authors do mention that "one-time PCR positivity is not a measure of cumulative infection", the discussion then goes on to admit the imperfect sensitivity of serology (as a measure of persistent infection), and seemingly uses PCR-based observations as the comparison standard after correcting for serology bias. It seems clear that the sensitivity of HPV detection (from serology) can have an appreciable effect on the outcome of the analyses. As such, the authors might consider quantifying the degree of this imperfect sensitivity (e.g. against some [PCR?] gold standard?), and how exactly the serology analyses were "quantified and corrected" (perhaps as an addition to the supplementary Laboratory Analysis).**

**Re:** We have carefully evaluated the impact of the less than 100% sensitivity of serology to detect cumulative HPV exposure. A validation study of this method of serology’s ability to measure cumulative HPV exposure has previously been conducted and reported by Artemchuk et al (reference 25), using PCR as a ‘gold standard’. However, as we also highlight in the discussion (page 16, lines 7-9), PCR positivity is not a measure of cumulative exposure. Thus, we have used these estimates of sensitivity and specificity only as a starting basis and have conducted probabilistic bias analysis (and not simple bias analysis) which accounts for the fact that there is a degree of uncertainty and error in these values. For further clarification, we have also now included a table (Table S1) of the sensitivity and specificity parameters used in the probabilistic bias analysis portion of the statistical analysis.

**3. A major focus of the paper is on "herd protection", given its emphasis in the abstract's motivation and conclusions, in particular the statement that "...when vaccination coverage is moderate, only gender-neutral vaccination results in herd protection against both HPV types 16 and 18". The directly relevant results shown in Figure 4 do seem to suggest a statistically-significant reduction in seroprevalence of HPV (from ~30% to ~23%) for gender-neutral (Arm A) vaccination, compared to only a marginal reduction for girls-only (Arm B) vaccination. However, the question is whether such a reduction would qualify as "herd protection". The authors might consider discussing the criteria for herd protection to be validated (i.e. if the seroprevalence reduction were to say 27% instead of 23%, would there still be herd protection?)**

**Re:** Herd protection is defined in the introduction on page 5 line 9, and is measured as the degree of decrease in HPV prevalence/incidence (unattributable to random or systematic error) among the unvaccinated in the post-vaccination era.

**Moreover, the results from Figure 4 appear to imply that the declaration in the Discussion section that "...the gender-neutral vaccination strategy with its superb impact on both HPV16 and 18 overcomes the obstacle of suboptimal girls-only vaccination coverage", might be slightly exaggerated.**

**RE:** We have now revised the passage in the discussion highlighted by the reviewer to tone down the strength of the statement (page 15, line 16). **4. Given the recommendation on aggregate population-level herd protection, it might be appropriate to have an idea of the movement dynamics of the study participants, if possible. In particular, did they tend to stay within their original communities (which determined the Arm they belonged to), or was movement between communities common? This is especially since it is stated that "...exposure to the indirect effects of HPV16/18 vaccination is defined as residing at the time of sample donation in one of the community-randomized HPV vaccination trial communities", which appears to implicitly allow for such movement. A brief discussion of how such movement (if prevalent) could inform the recommendations would be appreciated.**

**Re:** In this trial the intervention was implemented on the community level, in a cohort of communities. Thus, whoever resides in given trial community at the time point of outcome measurement is included in the outcome measurement. However, given that our study population was comprised of pregnant females under the age of 23, it is likely that this is the portion of the population which is least likely to move to a new community, which minimises the movement and does not really allow for the suggested discussion. **5. While the analyses were adjusted for maternal smoking, which is mentioned to be consistently higher in the control Arm C communities, this gives rise to the question of whether smoking is expected to have any direct effect on HPV/cervocal cancer prevalence. Moreover, how exactly were these statistical adjustments (including random/systematic error introduced in Table 1) implemented?**

**Also, since it is stated that "...all estimates were adjusted for community-wise self-reported smoking", the authors might comment whether omitting the adjustments produces similar conclusions in any case. This is especially since Figure 5 suggests that adjusting for smoking has caused Arm B (girls-only vaccination) to demonstrate higher HPV seroprevalence ratios compared to Arm C (the control).**

**RE:** The analyses were adjusted for community-level self-reported maternal smoking, as a surrogate of community level differences in general risk-taking behaviours which may account for differences in the incidence of HPV irrespective of community-wise vaccination, as described in the methods in page 9 (lines 4-6). The primary analysis adjusted for maternal smoking in the log binomial generalised estimating equation (GEE) model as described in the first paragraph on page 11. When conducting the sensitivity analysis accounting for systematic error, maternal smoking was not adjusted for as also indicated in the legend of table 1.

Regarding the second point, and the example raised regarding the impact of adjusting for community-wise self-reported smoking, in figure 4, the crude unadjusted seroprevalence is displayed which shows that there is a slight increase in HPV16 in the post-vaccination era in Arm B (more so than observed in Arm C). This the source of the (non-significantly) increased estimate highlighted from figure 5. These differences were within the error margins, suggesting that they are explainable by random error. **6. The population numbers in Figure 2 may be a little off. In particular, pre-vaccination total N=4006 is stated at the beginning, but the sum of the three Arms in the next step, 1326+1347+1334 is 4007. Similarly, post-vaccination total N=4016, but the sum 1331+1344+1340 is 4015.**

**RE:** We would like to thank this reviewer for meticulously noticing this disparity and for drawing our attention to it. We have checked our datafiles and corrected the Ns accordingly with the correct values (page 12, lines 6-7, figure 2).

**Additional minor comments follow:

7. It is stated in the Introduction that "Furthermore, in our community-randomized trial, gender-neutral HPV vaccination has provided significantly stronger herd effect against HPV18/31/33/35...". It might be clarified that this is from analysis performed in previously-published studies.**

**RE:** To clarify that this finding was from a previous published analysis using a different outcome measure we have referenced the previous studies (references 8 and 9) and have revised the text of this sentence (page 5, line 20).
 **8. The HBV abbreviation seems to appear without an initial definition (hepatitis B); it might also be relevant to mention that the HBV vaccine shows no clinically-relevant interference in antibody response to HPV antigens, if true.
Re:** Thank you for drawing attention to this, the HBV abbreviation has now been defined as hepatitis B virus on page 8, line 10.

**9. This sentence under the Statistical Analyses subsection might be reworded: "To evaluate the extent of exposure among the study population of pregnant females, where The exposure is defined as the indirect exposure to HPV16/18 vaccination due residing at the time of sample donation in one of the communities of the community-randomized trial".**

**Re:** Thank you also for bring our attention to this. We have now revised this passage so that it is more coherent (page 10, line 6). **10. For the sentence "Particularly the HPV16 seroprevalence was significantly decreased in the Arm A communities in the post-vaccination era compared to the pre-vaccination era (22.1% versus 17.4%)", the percentage figures might appear in the same order as the eras they reference (i.e. 17.4% versus 22.1%)**

**Re:** Again, thank you for noticing this discrepancy, we have now revised this sentence so that the percentages appear in the order that they were referenced (page 13, line 3).

-----------------------------------------------------------

**Reviewer #2: This is a carefully designed and appropriate study utilising HPV seroprevalence in young pregnant women ( plus some age matched  males)  from communities in Finland from pre and post HPV vaccination eras to  deliver a randomised investigation to measure the potential  impact of gender neutral vaccination to achieve better herd immunity  The principle  conclusion is that when HPV vaccination coverage is moderate, only gender-neutral vaccination establishes herd protection against HPV16 and HPV18, among unvaccinated females. This manuscript  provides important support for the implementation of a gender-neutral HPV vaccination policy to achieve optimal vaccine effectiveness when obtaining a girls-only vaccination coverage of 90% is impossible. Previous studies have utilised HPV DNA PCR endpoints and have not seen such herd immunity impacts with lower coverage. This is discussed as likely due to this measure not reflecting cumulative infection and inability to distinguish persistent virus and transient infection.**

**RE:** Thank you very much for this review.


**Reviewer #3: In this study, Gray et al. use Finnish HPV serology data to examine whether gender-neutral HPV vaccination leads to more herd effects than girls-only vaccination in unvaccinated young women. The use of serology data presents a different and interesting way of assessing HPV exposure compared with most other herd effect studies which have used HPV DNA detection. Notable study strengths include the large sample size, high participant consent rate, the linkage of data with a large community-randomized HPV vaccination trial, and the inclusion of a sensitivity analysis to assess the likely impact of non-differential misclassification error. However, some notable limitations of the study include the low representativity of the sample (pregnant women <23) and limited ability to control for possible changes over time occurring in the background risk of HPV exposure (secular trends). While the topic is important and the study may offer some interesting insights, I have
some major comments regarding the analysis and interpretations:**
***       The authors estimate that HPV vaccination coverage is approximately 47-53% in the eligible female birth cohorts in the post-vaccination era. However, very few women were excluded in the flowchart due to previous vaccination in the post-vaccination era (14/1331 in arm A, 23/1344 in arm B, and 12/1340 in arm C). This suggests that the women in the Finnish Maternity Cohort up to 2016 are a population of women where HPV vaccine coverage is at best 1-2% (likely they skew older and include women who may have been missed by the community trial). The authors should consider whether it is plausible that there would be herd effects when vaccination coverage appears to be so low in the study sample**.

**RE:** Thank you for stating this concern. When linking the subjects from the Female Maternity Cohort to the HPV vaccination register, this was done twice. Firstly, prior to the extraction of the serum samples (as being unvaccinated was part of the inclusion criteria), and then again after the serum samples had been extracted. The flowchart details those samples which were excluded post- serum sample extraction, through this second linkage procedure, and does not include the subjects initially found to be ineligible (HPV vaccinated) before sample extraction during the first linkage. We have now revised the text in the methods so as to make this clear (page 7, line 16), and added the number of subjects which were ineligible for the study initially due to this first linkage in the results (page 12, lines 5-6). In figure 1 and figure S1, the birth cohorts which are included in the post-vaccination era are visualised; the 1992-95 birth cohorts which were directly subject to the trial are included in the post-vaccination era.

***       The declines in HSV-2 seroprevalence over time suggest that there may be declines in HPV seroprevalence over time that may not be entirely attributable to vaccination programs, possibly due to differences in age distribution or in sexual behaviours. I would have liked to see this included somehow in the analyses, perhaps through the inclusion of a time trend in prevalence common to all HPV types. A time trend could have been included through the use of a difference in differences analysis, for example. Currently there is little mention of this in the discussion and of the possibility that observed changes could be due to other trends over time.**

**RE:** We would like to thank this reviewer for their meticulous attention to detail. HSV epidemiology has been globally undergoing a change in the recent decades, and Finland is no exception. In genital Herpes cases HSV-1 has been increasing as the cause whilst HSV-2 has been decreasing, due to several factors; an increase in the incidence of oral sex, and the increased awareness regarding the oral-to-oral transmission of HSV-1 which has resulted in a lower incidence in early childhood, thereby leading to an increased susceptibility to HSV-1 genital infection in adulthood. Further to which earlier studies have suggested that HSV-1 infection may be protective against HSV-2 acquisition particularly the infection is at the same site. Thereby the decrease in HSV-2 seropositivity over time is not entirely unexpected and may be independent of any changes in HPV incidence over the time frame.

However, we do agree with the reviewer that it could have been possible for HPV incidence to have changed over time even in the absence of HPV vaccination (to assume that it would be constant would certainly be ill-advised). To address this possibility, we compared the with-in Arm seroprevalence ratios between the Arms, as a ratio of ratios analysis. When conducting this analysis to take account of possible underlying secular trends, we still found that the HPV16 seroprevalence was decreased after gender-neutral vaccination (RPR=0.78, CI 0.64-0.95). Discussion of the possible impact of secular trends accounting for the observed change in HPV incidence has been further clarified in the discussion on page 18, lines 1-5.

***       The study sample is meant to be restricted to unvaccinated women. We need more information on how the authors identified "HPV-vaccinated" women based on their antibody levels, this is not very clear currently.**

**RE:** Thank you for raising this point, we have now revised the passage in the methods, describing how HPV vaccinated women were identified, so as to increase clarity (page 7, lines 13-20).

**Other comments:
*       I personally find it problematic and stigmatizing to label HSV-2 seropositives as a "sexual risk-taking core group" given how common HSV-2 is in the general population. Please consider rephrasing this in the paper as women who are likely to have had higher exposure to STIs, which is less judgmental.**

**RE:** Thank you for this comment. We have now rephrased the passage in the methods on page 9 (lines 6-7) with this comment in mind. In this paper we attempt to identify the core group (the epidemiological term for the group which have a high contact rate, and which tend to assortativity), as from the epidemiological perspective the production of herd effects in this group may differ to that of the general population. HSV-2 seropositivity is well established as a method to identify the individuals with-in the population which have a high number of life-time partners in multiple different populations. We therefore utilise HSV-2 seropositivity as a proxy marker of core-group membership.

***       The authors claim there is little difference in age distribution pre- and post-vaccination. However, small differences in age could have a big impact on HPV seroprevalence given the large expected increase in HPV prevalence with age during the late teens. It would be helpful to give the mean age of women pre- and post-vaccination or include a table of the age distribution by era in the appendix. Is there any reason that age was not adjusted for in the log binomial model?**

**RE:** There is no (material) difference in the mean age between vaccination eras nor Arms. The second table in the supplementary appendix displays the age distribution by vaccination era and Arm (Table S3). To further enhance this, we have now also included the mean age (plus the standard deviation) (Table S3).

***       The authors mention that pregnant women <23 are likely to be a more sexually active group, given that the average age at pregnancy is 29. The authors may want to mention that herd effects are expected to be lower and harder to detect in more sexually active groups, so their estimates are likely to underestimate herd effects in the general population.**

**RE:** Indeed our study may be limited in it’s generalisability to the general population as described in the discussion on page 17 (last paragraph). This is why we are studying both the general population and the core-group. The estimates of core-group specific herd effect in this study population were also notably reduced (contrary to the expectation raised in the above comment). Thus we prefer to err on the side of caution and not to suggest as to the direction of this bias at this point whilst there is still not an established large body of empirical evidence documenting the differential vaccine induced herd effect in the most sexually active group.

***       The serum samples were tested for more HPV types than were presented (39, 51, 52, 58, 59, 66, 68, 73). Why not present the results for these types, which are not expected to be affected by vaccine cross-protection? This would give further information as to whether there are time trends in HPV seroprevalence over time that are not attributable to vaccination.**

**RE:** In this paper we focus on the vaccine-covered HPV types, which includes the HPV types which are specifically targeted by the bivalent HPV vaccine, and those which the vaccine has been documented to provide cross-protection against. An evaluation of the trends in the incidence of these other non-vaccine HPV types has been conducted to evaluate the occurrence of vaccine-induced HPV type replacement and is about to be published shortly.

-----------------------------------------------------------

**Reviewer #4: This manuscript addresses the important topic of who to vaccinate against HPV. It is clearly written and makes an important contribution to our understanding of herd immunity. Herd protection was estimated via a repeated cross-sectional cohort study of all pregnant women under the age of 23 within thirty-three communities participating in a community-randomized HPV vaccination trial. Cumulative infection in unvaccinated patients was estimated using L1 VLP serology.  Communities were then randomized to one of three trial Arms: Arm A, gender-neutral HPV vaccination; Arm B, girls-only HPV vaccination; or Arm C, no HPV vaccination. This is a massive effort in a well followed nordic population.

The % of patients vaccinated within each cohort is hard to decipher as you read the manuscript. Please make this more clear in the text.**

**Re:** Thank you for stating that this was hard to decipher for the reader. To address this, we have now added an additional passage to the methods, to clearly state the community-wise vaccination coverage implemented via the community-randomised trial (page 8, lines 17-21).

**No estimate of the stability of the populations (i.e. how much intermingling) was provided, and should be.**

**Re:** As mentioned in response to reviewer 1 (their 4^th^ point) in this specific population of pregnant females which are still under the age of 23 years old, they are likely the portion of the population in these communities which are the least likely to move from community to community (in comparison to their non-pregnant counterparts).

**In Figure 4, the drop in HSV2 seropositivity in Cohort B was as profound and significant as for the drop in HPV16/18 seropositivity in Cohort A. Please explain as this seems to call into question the main findings of the paper**.

**Re:** Thank you for reviewing this manuscript with such a keen attention to detail. As also mentioned in response to reviewer 3, HSV epidemiology has been undergoing dynamic changes globally in the recent decades, due to several factors, including changing sexual behaviours (increased incidence of oral sex), a decrease in HSV-1 acquisition orally in childhood resulting in increased susceptibility to HSV-1 genital infection in early adulthood and the dynamics between HSV-2 and HSV-1 when at the same site of infection. HSV-2 epidemiology is therefore complicated and may be independent of possible counterfactual changes in HPV seroprevalence that would have occurred if there had been no HPV vaccination implemented.

However, even if there had been no change in HSV-2 serology post-vaccination, it would still be a faulty assumption to assume that HPV serology in Finland would have remained rigidly constant over time in the absence of vaccination. This is why we have also conducted the same analysis in the Arm C communities which were randomised to receive no HPV vaccination. When comparing the intervention Arm seroprevalence ratio estimates to that found in Arm C to take account of such possible secular trends, we still found HPV16 to be decreased after gender-neutral HPV vaccination (figure 5, in the Methods page 11, lines 7-10, in the Results page 14, lines 3-7, and further discussed in the Discussion page 15, line 5 and page 18, lines 1-5).

**They show that the HPV16 and HPV18 seroprevalence was decreased in young unvaccinated women after gender neutral vaccination, but with single gender vaccination under these conditions of low vaccination rates. This provides a demonstration of herd immunity, but is perhaps not all that surprising given that twice as many people in the gender neutral vaccination cohort were vaccinated. Nevertheless it shows the important message that when only low vaccination rates can be achieved in girls that there is value in vaccinating boys to improve protection of girls through herd immunity. It is also highly likely that boys will also receive direct health benefits themselves through prevention of anogenital cancers and head and neck cancers, and should be strongly urged anyway for reasons of equity.
Do you expect the impact of herd immunity to grow as the population ages?**

**Re:** We expect the impact of herd protection to further increase in the consecutive birth cohorts since HPV vaccination implementation as the transmission tends to be in the direction of the older to younger cohorts, as discussed in the discussion in the first paragraph of page 17, lines 1-7 (Vänskä et al, DOI: 10.1093/infdis/jiaa099).

**Is it reasonable to consider looking at the impact on CIN2/3 rates in the medical record as well as seropositivity?**

**Re:** It is reasonable to look at the impact on CIN2/3 rates in these cohorts. However, this will be conducted in the future as currently the females are still too young (the oldest cohort from this trial being only aged 27-28 years old currently).
-----------------------------------------------------------

Thank you sincerely for your consideration.

Yours Respectfully,

Penelope Gray and Prof. Matti Lehtinen

(Tampere University, and Karolinska Institutet)
