# Supplementary material for: Human papillomavirus seroprevalence in pregnant women following gender-neutral and girls-only vaccination programs in Finland: A cross-sectional cohort analysis following a cluster randomized trial
Source: PLoS Med. 2021 Jun 7;18(6):e1003588. doi: 10.1371/journal.pmed.1003588 (PMC8216524; doi:10.1371/journal.pmed.1003588)
Supplement: Attachment — Submitted filename: Response to Reviewers.docx [file pmed.1003588.s015.docx]

Comments from the Academic Editor:
The study is based on excellent data sources and a well-known and important HPV vaccine trial, with record linkage to a national cohort of and serum databank for pregnant women. Herd effects of HPV vaccination are a valid and useful outcome to examine through further analyses of the parent trial. But the revised version of this manuscript does not give enough information to understand motivation, planning, analysis plan or results adequately. After reading detailed and thoughtful comments of the reviewers, the actual changes to the manuscript are very limited. As one reviewer says, you need to go back to all the previous studies to piece things together.

*We are, of course, willing to further revise our manuscript to address the above comment: “…the actual changes to the manuscript are very limited.” However, to be on the same page we*

*repeat the two reviewer comments that were available from the editorial office concerning our revised manuscript (version May 2020).*

*Reviewer 1. “We thank the authors for considering the points raised in our previous review. They have largely been addressed, and only a few suggestions remain:..”*

*Reviwer 3. “Great work by the authors. Remaining comments:..”*

The paper is hard to follow for three main reasons. First, this report doesn’t explain well enough **how this particular analysis fits in with the original trial,** or the subsequently described plan to examine type replacement. Second, there are **inconsistencies between this paper and the analysis plan** that was provided. Third, it is not particularly easy to see **how herd immunity is being defined and measured.** I have a number of requests for clarification and suggestions.

*The three above points are now answered in the Introduction: 2^nd^ paragraph 2^nd^ sentence (first point), 2^nd^ paragraph 1^st^ sentence (third point), and end of the 3^rd^ paragraph, 3 last sentences. Please, note that we prefer to talk about herd protection or herd effect not herd immunity, not to confuse uninitiated readers since no real individual immunity in the unvaccinated is being established.*

General comments
1.      Explanations and context: In the introduction, please explain the rationale for this study more fully, as it relates to the original objectives of the trial. The clinicaltrials.gov entry doesn’t mention type replacement or herd effects as primary or secondary outcomes, but the study design was reported as an RCT. The original RCT (as presented in ref 18) included modelling and provision to examine herd effects within the trial population, but did not specify additional analyses. Explain more clearly what the previous publications (esp refs 8 and 9) did and did not address about herd effects (e.g. lack of herd effects shown for HPV16 using HPV DNA positivity).

*Please, see above and revised paragraphs 2 and 3 of the Introduction.*

2.      Without a protocol or statistical analysis plan written in advance for this particular analysis, it is hard to see whether the findings here showing support for a pre-defined hypothesis, or are exploratory and hypothesis-generating. In the introduction, please explain how the plan to assess herd effects of gender-neutral vaccination developed. The data from the Finnish Maternity Cohort (FMC) are mentioned in ref 18 for planning the trial, but not that they will be used in future. Explain how this study fits into the study analysis plan provided – that plan does not mention anything about measuring herd effects. The authors say they needed to find a herd effect before investigating type replacement. In this case, we would expect to see an analysis plan for investigating the herd effects before the analysis plan for type replacement. There is a new publication, which was not mentioned in the previous revisions, Gray P, et al. Int J Cancer 2020. Is this the manuscript that
used the analysis plan? Please clarify, in the manuscript.

*Thank you for stating these concerns. The presented study plan concerning serologically evaluating the two sides of HPV16/18 vaccination impact describes type-replacement as its primary hypothesis. This, however, requires creation of an ecological niche by the vaccination created herd effect/herd immunity, most notably with the gender-neutral vaccination. The randomised design to prove this we have published already in 2003 (with associated modelling published in 2006 and 2008) now duly described in the Introduction, and carefully documented in the community-randomised trial study protocol (enclosed). In the Introduction we now raise the question whether the demonstration of herd effect against HPV16 could have depended on the detection method (PCR vs. serology) used. The presented (type-replacement) study plan should have had a co-primary objective: Demonstration of HPV16/18 herd effect and Filling the created ecological niche* *by non-vaccine HPV types. The fact that only the latter was explicitly outlined does, however, not mean that also the herd effect hypothesis had not existed primarily (published for the first time already in 2003, reference 7a1). In addition to the Introduction, this is now described in detail at the beginning of page 8. In summary, our study is not hypothesis generating but testing the original (2003 published) hypothesis by modern HPV serology in a population-based setting. As noted in the end of the Introduction this is the natural counterpart of the serological type-replacement study published (Gray et al. Int J Cancer 2020).*

3.      Given these limitations, I think that the title and conclusions (as stated in the Abstract and end of the main text) are somewhat overstated. The second bullet point of ‘What do these Findings Mean? in the Author summary is more appropriate as a conclusion.

*Thank you for this comment. The title and conclusions made from the results have now all been revised with this in mind.*

4.      Methods, study design could be clearer. The first two pages are confusing because they jump between the parent trial and the Finnish Maternity Cohort.

*Thank you for noting this – the order of presentation is revised as suggested.*

4a.     Please state the hypothesis, if there was one, or if not that these are exploratory analyses.

*The primary hypothesis is now stated at the beginning of page 8.*

4b.     Please state the primary outcome. I couldn’t find definitions of which was the primary and which were secondary outcomes. Similarly, results were not reported according to primary or secondary outcomes.

*The Methods and Results sections have been revised. The primary and secondary outcomes explicitly read at the beginning of pages 10 and 11 (Methods), and on the two subheadings of page 14 (Results)*

4c.     Fig 1, please add a first level to the flow chart to show how the cohort studied in this trial is related to the parent trial, including total numbers in the communities in each arm.

*We have now made added an additional part to the top of the flow chart (Figure 2) to show how this study is related to the community randomised HPV trial, and how it is related to the source population.*

4d.     Statistical analyses. The study is an analysis of a cohort nested in an RCT. Please describe how you measure follow-up and account for losses to follow-up. Please compare the characteristics of cohort participants to those of the female population of the same age in the trial communities.

*Yes, the study is “nested in a RCT” which is by definition a cohort in which the exposure has been randomised, and yes, the biobank is officially called the “Finnish Maternity Cohort”. However, in our study the sampling of FMC donors was cross-sectional; all females under the age of 23 who had donated samples during their first pregnancy to the FMC biobank between 2005 and the end of 2016. There are no serial samples from the same individual, and there is no active follow-up of* *individuals in which loss to follow-up could occur. In the timeline between exposure and outcome measurement, the participant is selected into the study at the time of outcome measurement (i.e. sample donation). It is an example of what is known as a “Cross-sectional cohort study” (see reference 14, by Hudson et al), which in this case is nested in a cluster randomised trial. To call it anything else would be wrong.*

*Moreover, if we consider this study at the cluster level; as we have not lost any of the communities in our study between 2005 and 2016, there is no loss to follow-up in this sense.*

4e.     The analysis plan provided says that the pre-vaccination period is 2005-2007 and the post-vaccination period is 2008-2016. In the analyses here, the pre-vaccination period is 2005-2010. But vaccination was given in 2007-2010, so these years are part of the vaccination and post-vaccination period. Please explain.

*Explained of page 10, end of 1^st^ paragraph with pertinent references.*

5.      Herd effects. These can be hard to conceptualise and understand.
5a.     The authors now give a definition of herd protection. Could they give the full definition in the text that they gave in the response to reviewers?

*We have now given the full definition of herd protection/herd effect as previously described in response to the reviewers in the manuscript text, on page 11 .*

Could they also address Reviewer #1’s question about the relevant size of the difference?

What size of difference was hypothesised?

*The first reviewers’ previous question, which we received back in May, was as follows:*

Reviewer #1: “The directly relevant results shown in Figure 4 do seem to suggest a statistically-significant reduction in seroprevalence of HPV (from ~30% to ~23%) for gender-neutral (Arm A) vaccination, compared to only a marginal reduction for girls-only (Arm B) vaccination. However, the question is whether such a reduction would qualify as "herd protection". The authors might consider discussing the criteria for herd protection to be validated (i.e. if the seroprevalence reduction were to say 27% instead of 23%, would there still be herd protection?)”

*We apologise for not sufficiently addressing the reviewer #1’s previous question on the relevant size of the difference. As with many things in biology, the extent to which herd effect may manifest in the unvaccinated portion of a population is on a continuous scale. It is the opinion of the authors of this manuscript that to implement a specific cut-off, for example that at 20.9% reduction there is no herd effect, whilst at 21.0% there is herd effect, would be illogical. However, in light of this point we have now revised the manuscript so as to increase the emphasis on the magnitude of the observed decreases in seroprevalence post-vaccination. In the same line of thought, we have also changed the terminology so that in places where it previously said herd “protection” when discussing the results of this study, it now reads as herd “effect”, as the word “protection” has more connotations of complete 100% protection in comparison to the word “effect”.*

5b.     What was the vaccine coverage in the three arms? Is it possible that there is a greater effect in Arm A because overall coverage is higher? In Arm A, girls+boys coverage = 47.1% of girls + 19.5 of boys%. In Arm B, it’s 45.8% of girls.

*For a sexually transmitted infection, assuming heterosexual transmission, the impact from vaccinating 19.5% of boys comes from first order herd effect which prevents transmission from a vaccinated boy to a non-vaccinated girl and vice versa. Please, see our recent J Infect Dis (222:948-956, 2020) paper by Vanska et al. on the outlier-free analysis of the community-randomised trial.*

5c.     The authors report three different analyses, but these don’t seem to be comparable with respect to factors that are controlled for or stratified by. Please clarify these choices.

*The three different types of analyses: Comparison of seroprevalences by arm, Comparison of seroprevalence ratios within arm, and Comparison of seroprevalence ratios between arm are deliberately done to disclose the full impact of the different vaccination strategies over time (vaccination era).*

5c.i.   Indirect effect of ‘community-wise’ vaccination over the study period – absolute seroprevalence in pre- and post-vaccination periods – why not controlled for smoking, or stratified by HSV-2 status?

*We have added new tables to the supplementary appendix, where the absolute seroprevalence may be found also stratified by HSV-2 seropositivity.*

5c.ii.  Further assess indirect effect of ‘community-wise’ vaccination in the post-vaccination era – within-arm seroprevalence ratios, comparing post-vaccination and pre-vaccination (controlled for smoking – but we should see the crude ratios too)

*Crude ratios are now displayed.*

5c.iii. Overall indirect effect of gender-neutral and girls-only, compared with counterfactual –between-arm ratio of seroprevalence – ratio of ratios - ? controlled for smoking?

*As described in the methods and the figure legend, the Ratio of ratio is calculated using the well-known methodology of Altman and Bland, the with-in arm estimates used to compute the between Arm estimates are adjusted for community level self-reported maternal smoking. To make this clearer we have revised the methods on page 13, line 306.*

6.      Discussion, Interpretation according to gender-neutral strategy: Is there any information on sexual mixing between girls and boys who are vaccinated or not vaccinated? How would this affect the findings if there were assortativity according to vaccination status?

*The reviewer raises the possibility that HPV vaccine shots between ages 12 to 15 would have had assortative impact on sexual mixing during the next 3 to 6 years. To preclude this possibility our trial was blinded in Arms A and B where HPV vaccine was used, respectively for 90% of female and male and for 90% of female participants. In Arms A and B, HBV vaccinated comprised about half of the non-HPV vaccinated trial participants. In the control Arm, HBV vaccine only was used. Due to these facts, the blinding and ethical review board’s specific denial to combine sexual behaviour questionnaire data with other trial data at personal level the requested information is not available. Overall, there were no differences in sexual behaviour between Arms A, B and C at age 18.5, and we think that the suggested assortativity was negligible but have made a comment to study limitations.*

Specific queries (page and line numbers as in the marked up copy because there are none in the clean version of the pdf)

1.      ‘Community-wise’ – doesn’t make sense – should it be community-wide (across all communities)? Or community-specific (for a specific community)?

*We apologise for the previous lack of clarity. This phrase was used multiple times in different contexts. Thus on page 9, line 206, where it refers to the maternal smoking variable, we have revised this phrase to read “community-level”. On page 10, lines 232 and 233, where it refers to vaccination coverage we have revised the text to read “community specific”, as it refers to the vaccination coverage specific to each community. On page 10 and 11, lines 239, 247 and 251, we have revised the text to read “community-level”¨.*
2.      Arms A, B and C. It’s hard to remember which arm is which. In some parts of the manuscript, it would be easier to refer to the gender-neutral arm of the trial (maybe with Arm A in brackets).

*Thank you for drawing attention to this, the manuscript has now been revised accordingly on page*

3.      P8, line 193: why ‘1982 and younger birth cohorts’? Should this be 1992?

*The eligible females in the study were all those comprising the female maternity cohort aged under 23 years old from 2005 until the end of 2016. In 2005 the oldest birth cohort under 23 years old were those born in 1982. Therefore, the eligible birth cohorts in the sampling frame were those born in 1982, 1983, 1984, 1985, 1986, 1987, 1988, 1989, 1990, 1991, 1992, 1993, 1994, 1995, 1996, and onwards.*

4.      P10, line 226: ‘due residing’ doesn’t make sense.

*Thank you for pointing this out, we have now revised the text to include the missing word (“due to residing”).*

5.      P10, line 231-2: ‘weighted to the birth cohort distribution found in each year…’ ‘distribution’ of what? Age? Sex?

*The* *coverage is weighted according to the distribution of birth cohorts found in each year of sampling. We have now revised the highlighted passage with the aim of making this clearer.*

6.      P12, line 282-3: HSV-2 seroprevalence – please refer to Fig 4.

*We have now made reference in the text to figure 4.*

7.      P13, lines 306-8: Figures in Table 1a and text don’t match.

*The figures in table 1a and in the text do match. However, whilst examining the text and tables for such inconsistencies, we noticed that there was an inconsistency between the figures in table 1b and the text. Thank you very much for drawing our attention to this. The figures in the text are correct, we have now rectified table 1b.*

8.      Fig 3. Why is there no shading in the years 2007-2010, when HPV vaccination was offered as part of the trial?

*There is virtually no shading in the years 2007-2010, as this figure displays the vaccination coverage among the birth cohorts represented in the Finnish maternity cohort, and although PV vaccination was offered in the years 2007-2010, it was only offered to the 1992-95 birth cohorts, who were at the time early adolescents (mostly aged between 13-15 years old). Therefore, although there are a small number of females who were pregnant from the 1992-95 birth cohorts between 2007 and 2010 (N=28 in Arm A and N=26 in Arm B), they were so few, that the slight change in vaccination coverage only just becomes visible in 2010, ranging from 2.7% (in Kokkola) to 5.2% (in Kemi).*

9.      P15, line 346, ‘randomized trial evidence’ – please qualify this, given that the study population is a subset of the women in the communities.

*We have removed the phrase “randomized trial” from this line.*

10.     P15: please explain why Chlamydia trachomatis as a marker of high sexual partner change (core group membership), would not be associated with a herd effect against HPV16 but HSV2 seroprevalence would?

*We have not stated that the different measures of core-group membership are the reason for the different observations of HPV16 herd effect. Chlamydia trachomatis current infection versus HSV2 seropositivity are of course two distinctly different measures, which are used as a proxy of current vs ever core-group membership. It is true that previously we did not observe HPV16 herd effect in the core group in a study where we used Chlamydia trachomatis current infection as a proxy of core-group membership, as is mentioned in the manuscript. However, we do not presume to state that it is because of the different measures of core-group membership. There is insufficient evidence for such inference. It is important to remember that in these two studies, firstly, different measures of HPV 16 infection were used (PCR positivity in the previous study, and serology in this one)(as discussed in detail in the discussion), and secondly, different birth cohorts were included in the two studies (as also discussed on page 19). Both of these factors are likely to have impacted the observation of HPV16 herd effect among the core group irrespective of the core-group identification proxy used*.


Requests from the editors:

1- Thank you for updating the study design to cluster-randomized. However, following our email correspondence, it is now clear that this was a cohort study based on data from a cluster-randomized trial. Please update the Title and all other mentions of the study design throughout the manuscript to reflect this.

*We have now revised the title in accordance with the editor’s 5^th^ request. Similarly, we have revised the manuscript throughout.*

2- Please also include the prospective analysis plan (from our email correspondence) as a supplemental file, referenced from the Methods section. Any changes in the analysis-- including those made in response to peer review comments-- should be identified as such in the Methods section of the paper, with rationale.

*The prospective pre-analysis plan has now been added as a supplemental file. The pre-analysis plan has additionally been referenced in the methods section, and specific changes in the analysis plan have been specified and the rationale given on page 11, lines 251-254.*

3- Thank you for including the Cluster Trials CONSORT extension. However, as an observational study, it is more appropriate to report according to the STROBE Guideline, and include the completed STROBE checklist as Supporting Information. Please add the following statement, or similar, to the first section Methods: "This study is reported as per the Strengthening the Reporting of Observational Studies in Epidemiology (STROBE) guideline (S1 Checklist)." and remove the CONSORT checklist (and its mention in the Methods)

*We have now removed the Cluster Trials CONSORT extension and the mention of the CONSORT checklist from the Methods section. We have now added the statement as directed in the Methods on page 9, lines 211-213, stating that this study is reported as per the STROBE guideline and have uploaded the completed STROBE checklist as supporting information.*

The STROBE guideline can be found here: <http://www.equator-network.org/reporting-guidelines/strobe/>

When completing the checklist, please use section and paragraph numbers, rather than page numbers.

4- Please describe briefly the ethical, legal, or contractual restriction that prevents you from sharing the deidentified dataset publicly. Please also note that a study author cannot be the contact person for the data. Please provide a different contact for data access, such as a member of your institute’s data or ethics committee.

*We have now revised the data availability statement to briefly describe the ethical and legal restrictions which prevent us from sharing the deidentified dataset publicly, and have provided the appropriate contact information for data access.*

5-Please revise your title according to PLOS Medicine's style. Your title must be nondeclarative and not a question. I recommend "Human papillomavirus seroprevalence in pregnant women following gender neutral and girls-only vaccination programs in Finland: a cohort analysis following a cluster randomized trial.”

*We have revised the title so that it is nondeclarative and not a question, thank you for the suggestion.*

6- Throughout the manuscript, please replace “subjects” with “participants”.

*We have now revised the manuscript so that it reads as “participants” rather than “subjects”.*

7- Abstract Background: Please be more specific about what you mean by “medically most important HPV type”, and cite the actual reproductive number instead of “high”.

*We have now revised the text in the abstract background so as to be more specific regarding the “medically most important HPV type”. We would have cited a previously estimated reproductive number, however basic reproductive number is a value which is population dependent, therefore, to cite a number in the abstract might be misleading to the reader.*

8- In addition to stating the number of communities in each arm, please also include the number of participants in each arm when first describing the study in the Abstract.

*The number of participants in each arm has been added to the abstract, on page 2.*

9- In the Abstract and throughout the Results, please report all main outcomes (i.e., seroprevalence differences for all HPV types).

*The abstract and results (page 15 and 16 of the marked copy) have been revised accordingly.*

10- Thank you for mentioning a study limitation at the end of the Abstract Methods and Findings. Please make this a separate sentence(s), and include other relevant study limitations (e.g., generalizability outside Finland, potential effects of secular trends in sexual behaviors).

*We have revised the abstract and included description of relevant limitations of the study.*

11- Abstract Conclusions: Please address the study implications without overreaching what can be concluded from the data; the phrase "In this study, we observed ..." may be useful.

*We have revised the abstracts conclusions, so as to avoid overreaching what can be concluded.*

12- Additionally, in the Abstract Conclusions, please interpret the study based on the results presented in the abstract, emphasizing what is new without overstating your conclusions. Perhaps, “These findings suggest that gender-neutral vaccination can facilitate the control of HPV16…”

*The abstract conclusions have been thus revised, so as to avoid overstating the conclusions.*

13- Author summary point 3, please edit to: “Achieving herd protection for HPV16 requires greater than 80% girls-only vaccination coverage, a level that has not been achievable in many countries.”

*We have now edited the third author summary point to read, “Achieving herd protection for HPV16 may require greater than 80% girls-only vaccination coverage, a level that has not been achievable in many countries.”*

14- Author Summary point 4 (“Evidence-based solutions to this problem are required...”) should be removed and replaced with the study question.

*The author summary point commencing with “Evidence-based solutions…” has now been removed and replaced with the study question.*

15- Author Summary point 7 (“Only among the unvaccinated females...”) is phrased a bit awkwardly, consider editing.

*The sentence highlighted from the author summary has now been rewritten (page 4).*

16- The last sentence of the Introduction could be split into two sentences to improve clarity.

*The last sentence of the introduction was indeed lengthy and lacking readability. We have now revised the text.*

17- For all observational studies, in the manuscript text, please indicate: (1) the specific hypotheses you intended to test, (2) the analytical methods by which you planned to test them, (3) the analyses you actually performed, and (4) when reported analyses differ from those that were planned, transparent explanations for differences that affect the reliability of the study's results. If a reported analysis was performed based on an interesting but unanticipated pattern in the data, please be clear that the analysis was data-driven.

*The primary hypothesis and means to analyse it has been given in the Methods section under the sub-headings: Study design and Statistics. No data driven analyses were performed - the step-wise analyses were done to disclose different aspects of herd effect over time.*

18- Please remove trademark symbols (e.g., CervarixTM, Engerix®-B).

*The trademark symbols have now been removed.*

19- Thank you for including vaccination coverage. Please include the numerators and denominators that go along with each percentage. Additionally, this seems like it should be in the Results rather than Methods?

*The two vaccination coverages to which the above comment is referencing, are calculated as the mean of the 11 individual community specific coverages (from the 11 communities in the Arm to which the estimate belongs). This is indicated in the text by the phrase “mean community-level vaccination coverage”. The estimates do not therefore have a numerator and denominator. We have already evaluated and presented the community-level vaccination coverage among the study population in detail in the results and figures. The two vaccination coverage percentages presented in the methods are not results of this study but are describing the design of the parent cluster randomised trial, therefore it belongs in the methods.*

20- Please provide a table showing the baseline characteristics of the study population.

*A table showing the baseline characteristics of the study population is presented in the appendix (Table S3).*

21- Regarding the point (made by reviewers 1 and 4) about the likelihood of intermingling among the study population, your response should be noted in the manuscript text, in case readers also have the same question. Also, is there a reference you could point to on this point?

*We have now added to the manuscript a discussion on the possibility for inter-community movement and the possible bias it may introduce into our estimates on page 18, lines 419-424.*

22- Figure 5, the color legend is over top of the bottom ratio numbers.

*The colour legend of figure 5 has been moved to its correct position.*

23- Please integrate your response to Reviewer 4’s 4th point (about the HSV seroprevalence) into the main text, as readers may have the same question.

*We have now integrated our reply regarding the 4^th^ reviewers point about HSV-2 seroprevalence into the discussion of the main text.*

24- Please include a table of absolute numbers of seropositive cases, in addition to the information on adjusted seroprevalence ratio contained in the current table 1.

The absolute numbers of seropositive cases are now included in table S4.

25- Please incorporate table S3 into the main paper.

*Table S3 is now incorporated into the main paper in the second paragraph of the results.*

26- Please begin the Discussion with a brief summary of what was done, before describing the results.

*Done as requested.*

27- The phrase “sexual risk-taking core group” still appears, please replace with a less stigmatizing phrase.

*We have now removed the phrase “sexual risk-taking core group” from page 15, line 344, and replaced it with the technical term “core-group”, which is widely used in academic literature to refer to this group.*

28- Please rephrase the final paragraph of the Discussion to limit the conclusions to the current study. E.g., “Our results suggest that when HPV vaccination coverage is moderate…”

*Done as requested.*

Comments from the reviewers:

Reviewer #1: We thank the authors for considering the points raised in our previous review. They have largely been addressed, and only a few suggestions remain:

1. The additional advantages of the new cumulative incidence methodology over the previous one-time PCR-positivity might be briefly mentioned at the end of the Introduction section, for readers to more quickly recognize the significance of the work. The relevant previous work might also be cited there.

*The introduction has now been revised so in order to highlight more the advantage of the new measure of infection in comparison to the previous studies (page 5 and 6).*

2. The (low) possibility of movement between communities might also be briefly mentioned as a possible limitation.

*We revised the discussion section of the manuscript to mention the possible movement of study subjects between the communities and the possible bias such movement may introduce.*

3. The addition of Table S1 is much appreciated. If possible, it might be briefly explained as to how the sensitivity/specificity used in the analysis, were derived from the primary assumption.

*In page 11, lines 255-262 of the main manuscript it describes how the sensitivity/specificity used in the analysis was derived from that primarily assumed.*

Reviewer #3: Great work by the authors. Remaining comments:
*       It is recommended in flow charts by CONSORT to include all those assessed for eligibility in the study and document reasons for exclusion from the study. Please include in the flowchart all women <23 in the Finnish Maternity Cohort in the communities, and indicate how many were excluded due to lack of consent and the 3498 excluded due to registry-linked HPV vaccination status by study period in Figure 2.

*The flowchart has been revised with the addition of a new section at the top, indicating the number of women under 23 years old in the trial communities from 2005-16, and subsequent exclusions.*

* Please indicate what cutoff was used when assessing HPV vaccination status through antibody titers.

*When assessing the HPV vaccination status through the antibody titres, we restricted this process only to the females from the birth cohorts eligible for vaccination via the national vaccination program (and catch-up), those born in 1998, 1999 and 2000. This was not performed via setting an algorithm with a specific cut-off and excluding all the samples which were above a set level but was rather done by eyeballing the data. It is well established that the bivalent vaccine (which is used in the national program in Finland) produces high antibody titres phenomenally higher than that developed in response to natural infection (Artemchuk et al, J Infect Dis. 2019). Thus all the samples which had such abnormally high HPV16 and 18 specific antibody titres from the three eligible birth cohorts were thus removed (N=24).*

*       They repeat the definition of exposure twice in the methods, this sentence could be deleted: "Exposure to the indirect effects of HPV16/18 vaccination is defined as residing at the time of sample donation in one of the community-randomized HPV vaccination trial communities."

*Thank you for drawing attention to this. We have revised the methods so that the exposure is only defined once.*
